# A comparison of floating catchment area parameters with applications to a dataset of clinics enrolled in a statewide child and adolescent psychiatric consultation program

**DOI:** 10.3389/fpubh.2025.1498819

**Published:** 2025-02-20

**Authors:** Jocelyn Hunyadi, Lara S. Savas, Kehe Zhang, Jeanette E. Deason, Ryan Ramphul, Melissa F. Peskin, Erica L. Frost, Cici Bauer

**Affiliations:** ^1^Department of Biostatistics and Data Science, School of Public Health, The University of Texas Health Science Center at Houston, Houston, TX, United States; ^2^Center for Spatial-Temporal Modeling for Applications in Population Sciences, School of Public Health, The University of Texas Health Science Center at Houston, Houston, TX, United States; ^3^Department of Health Promotion and Behavioral Sciences, School of Public Health, The University of Texas Health Science Center at Houston, Houston, TX, United States; ^4^Department of Epidemiology, Human Genetics and Environmental Science, School of Public Health, The University of Texas Health Science Center at Houston, Houston, TX, United States

**Keywords:** floating catchment area, spatial accessibility, kernel density, mental health, comparative analysis, access to healthcare

## Abstract

**Introduction:**

Healthcare resources are often crucial but limited, requiring careful consideration and informed allocation based on population needs and potential healthcare access. In resource allocation settings, availability and accessibility of resources should be examined simultaneously. The two-step floating catchment area (2SFCA) method has been previously used to evaluate spatial accessibility to healthcare resources and services, and to address health-related disparities. The 2SFCA methods have regained significant popularity during the COVID-19 pandemic, as their application proved crucial in addressing priority public health data analysis, modeling, and accessibility challenges. However, comprehensive comparisons of the 2SFCA method input parameters in the context of public health concerns in Texas are lacking. Our study aims to (a) perform a comparative analysis of 2SFCA input parameters on patterns of spatial accessibility and (b) identify a 2SFCA method to guide evaluation of equitable allocation of scarce mental health resources for children and adolescents in Texas.

**Methods:**

We used the Texas Child Psychiatry Access Network (CPAN) data to assess county-level, regional patterns in access to pediatric psychiatric care, and to identify areas to expand CPAN to mitigate access-related disparities. Using the 2SFCA method, we further compared accessibility patterns across two kernel density distance decay functions for 10 catchment area specifications.

**Results:**

As expected, spatial accessibility measures, such as the spatial accessibility ratio (SPAR), are sensitive to input parameters, particularly the catchment area. However, across all catchment area thresholds, two clusters of counties in southern and central Texas had particularly low accessibility, highlighting the opportunity for expanding the provider network in these areas.

**Discussion:**

Identifying areas with low accessibility can help public health initiatives prioritize regions in need of improved services and resources. The incorporation of additional data on supply capacity and care-seeking behavior would aid in the refinement of estimates for spatial accessibility at the regional level and within larger urban centers.

## 1 Introduction

Healthcare resources are crucial but limited, requiring careful consideration and informed allocation based on population needs. In the US, healthcare spending has consistently exceeded that of 38 other high-income countries, reaching 12.5% of GDP in 2000 and 17.8% in 2021 (1). Despite this substantial investment, the performance of the US health care system has been comparatively poor, ranking last among 11 high-income countries (2). The emergence of infectious diseases, such as COVID-19, further strains healthcare systems by escalating the demand for resources. In such challenging circumstances, maximizing access to healthcare services becomes paramount.

Barriers associated with access to healthcare have commonly been grouped into five dimensions: availability, accessibility, affordability, acceptability, and accommodation (3). Availability is often defined as the number of service or supply points, such as clinics or hospitals, while accessibility concerns the travel impedance between points of demand and points of service. In resource allocation settings, particularly during unprecedented events such as the COVID-19 pandemic or to address chronic priority health problems, availability and accessibility should be considered simultaneously. This combined measure is often referred to as spatial accessibility. In 2000, Radke and Mu (4) proposed the two-step floating catchment area (2SFCA) methodology to evaluate spatial accessibility to social services, building upon previous work by Shen (5) and Weibull (6). This method, later refined by Luo and Wang (7, 8), employs two core steps to measure spatial accessibility based on a travel impedance measurement, such as travel distance or time. Compared to previous methods, the 2SFCA utilizes a floating “catchment” that is not restricted by county or other geographical delineations. Since its inception, the methodology has been expanded, refined, and adjusted to create a family of spatial accessibility methods defined broadly as the floating catchment area (FCA) family. FCA methods have been used to assess spatial accessibility to health care facilities and primary care physicians (7, 9–13), care facilities for older adults (14), cancer care (15), pharmacies (16), parks and green spaces (17), and food stores (18). Since the COVID-19 pandemic, the FCA methods have gained substantial popularity, and have been utilized to assess access to testing sites, vaccination centers, and other services related to pandemic response (19–22). These analyses have aided in evaluating pandemic response strategies and guided decisions for optimizing resource allocation during such crises.

Despite the FCA family's popularity, there remains a significant knowledge gap in comprehensive comparisons between various FCA method parameters through the public health lens. One study compared the relative importance of distance decay (travel impedance) function selection and catchment area thresholds, but it lacked a public health focus and perspective (23). Another study by Wang et al. (24), compared three GIS-based accessibility approaches, including the gravity model from which the FCA is derived, to urban parks in Australia and the Netherlands. A recent study by Luan et al. (25), compared multiple measures of accessibility in New York City using the 2SFCA and Gaussian 2SFCA approaches, emphasizing health-related shortage areas. To our knowledge, no study has provided a comprehensive comparison of FCA accessibility parameters across Texas from a public health perspective. Therefore, our objectives were to compare county-level accessibility patterns from the 2SFCA approach across (i) two kernel density functions for travel impedance and (ii) 10 different catchment thresholds, indicating the maximum travel time or distance.

We used an early sample of data on clinics participating in a telehealth program designed to provide community-based Texas primary care physicians (PCPs) and pediatricians with mental health consultation services from child psychiatrists and other mental health professionals to meet the behavioral health needs of children and adolescents (26). We chose these data to illustrate a public health response to the ongoing rise in mental health problems in the US, especially among adolescents. According to a recent survey by the Centers for Disease Control and Prevention (CDC), the proportion of emergency department visits related to mental health issues increased by 24% among children aged 5–11, and by 31% among adolescents aged 12–17 from 2019 to 2020 (27). Further, social and economic factors, including socioeconomic status, insurance coverage, and stigma, have been previously shown to be barriers to mental health services and may further decrease access for vulnerable and underserved children and adolescents (28–31). With the dual objective of performing a comparative analysis and addressing the need for refined spatial evaluation methods to guide equitable allocation of scarce mental health resources for children and adolescents, our study contributes to the field of spatial accessibility research and its relevance in tackling critical healthcare challenges.

## 2 Materials and methods

### 2.1 Overview

**Figure 1:**
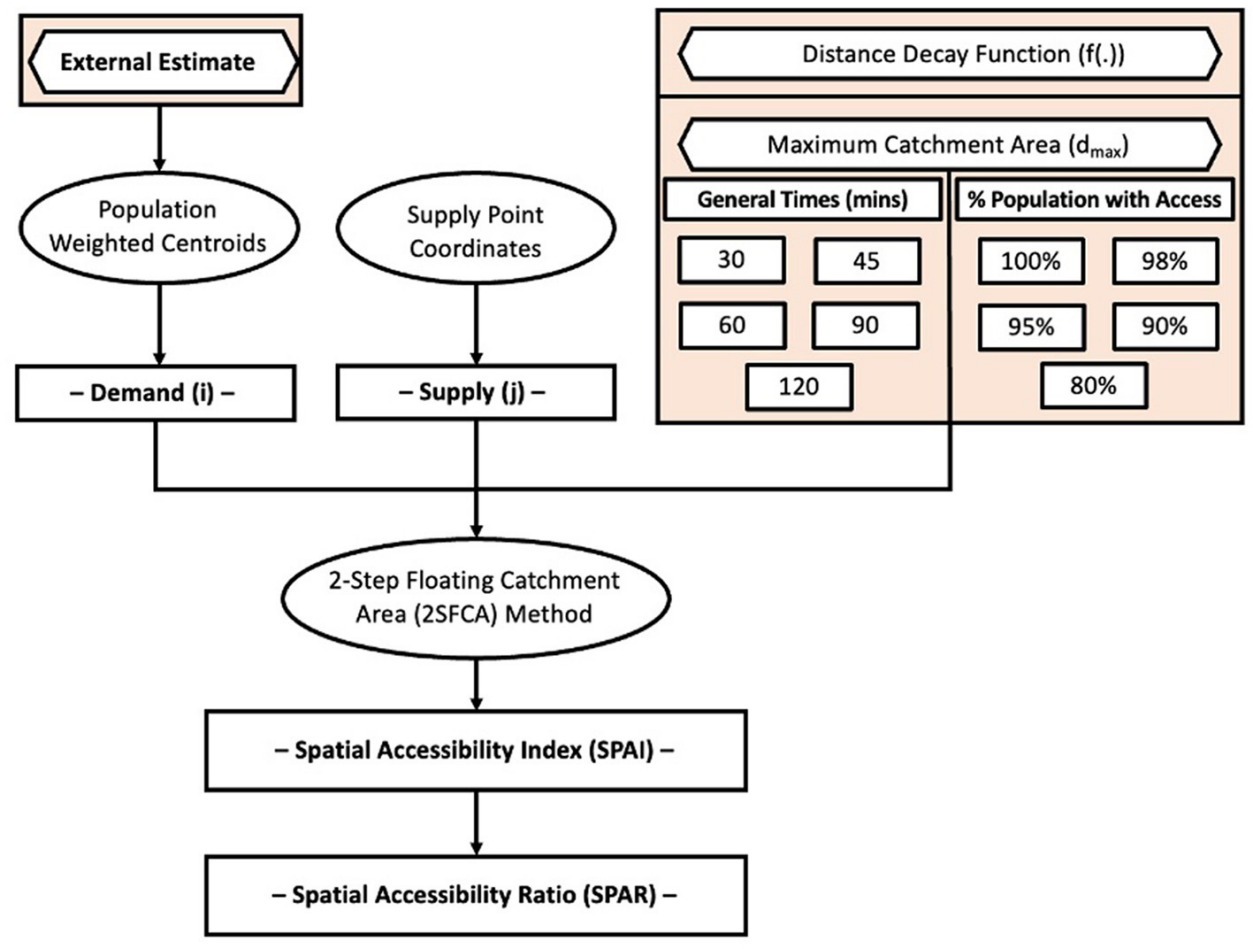
Methodological outline of the metrics and variable inputs for a typical 2-step floating catchment area (2SFCA) analytical algorithm.

We first provide an overview of the methodology used and modifiable parameters in the two-step floating catchment area (2SFCA) analysis, as outlined in Figure 1. The workflow highlights the four key adjustable inputs: (1) demand measure, (2) supply measure, (3) the construction of a maximum catchment area based on travel time or distance, and (4) the application of a distance decay function to account for travel impedance and supply-seeking behaviors. For our comparative analysis, we focus on adjusting the distance decay function and maximum catchment area.

### 2.2 Data

We used data on Texas clinics participating in a statewide child and adolescent psychiatric telehealth consultation program (initiated in 2020) to compare the 2SFCA approach with different kernel distance decay functions and catchment inputs to examine access to pediatric mental health care opportunities. The goal of this program is to connect community-based primary care physicians (PCPs) to health-related institution (HRI)-based psychiatrists for consults or advice on treating childhood and adolescent patients with mental health concerns (26). Integrated primary care models that link mental health specialists with PCPs have shown strong potential for improving access to mental health services and appropriate care for mental health problems (32, 33). In the first phase, the program aimed to enroll clinics within vulnerable populations or geographic areas with limited access to psychiatrists.

In this analysis, we included a list of participating clinics with signed agreements during the 1st year of program rollout (*N* = 1,707) (26). We only considered the clinic locations, as the initial phase of the program aimed to identify participating clinics to improve geographic coverage. Although additional information on the clinic's capacity (e.g., number of PCPs, etc.) will be incorporated as the program develops, it was not available at the time of analysis and thus not included here. We first geocoded the clinic location coordinates using the Google Geocoding API (34) and excluded three clinics outside Texas (Supplementary Figure 1A). We obtained county-level population estimates for children and adolescents (5–17 years old) from the 2017 to 2021 American Community Survey (ACS) (35) and population-weighted county centroids (in latitude and longitude) from the US Census (36).

For the demand estimation, we used the county-level ADHD estimates generated by Zgodic et al. (37). The authors used data from the National Survey of Children's Health (2016–2018), along with related area-level covariates and mixed effects logistic regression with post-stratification to create small-area estimates of ADHD prevalence (37). We defined demand as the estimated number of children and adolescents with ADHD, which is the product of the ADHD prevalence estimate and the corresponding county pediatric population. The study by Zgodic et al. (37) was conducted independently from the Texas child and adolescent psychiatric consultation program; ADHD diagnoses were obtained from survey data and small-area modeling and, thus, do not reflect active diagnoses found by clinics enrolled in the Texas program. Population-weighted centroids of each county were used as the spatial points of demand.

Additionally, we used the CDC's 2020 Social Vulnerability Index (SVI) and 2013 county-level Rural-Urban Continuum Codes (RUCC) from the US Department of Agriculture Economic Research Service (38, 39). SVI quantifies how social and environmental factors affect community resilience by assigning each county a rank from 0 to 1, with higher values indicating greater vulnerability. The SVI evaluates US counties based on 15 social factors across four themes: socioeconomic status, racial/ethnic minority, housing/transportation, and household characteristics. RUCC codes consider population size, degree of urbanization, and adjacency to a metro area to classify counties on an urbanization scale from 1 to 9, with 1 to 3 representing metro (urban) categories and 4–9 representing non-metro (rural) categories (Supplementary Figure 1). In this analysis, we included all Texas counties (*N* = 254) with corresponding RUCC data.

Prior to performing 2SFCA, we first constructed the Origin-Destination (OD) matrix, which is designed to quantify the travel distance or duration from a given demand (origin) point *i* to a supply (destination) point *j*. Each cell within the OD matrix represents a unique origin-destination pair. The resulting OD matrix is of *J* by *I* dimensions, where *J* is the number of destination points and *I* is the number of origin points. Travel distance and time between each unique pair was completed using ArcGIS Pro (version 3.1.1) with the StreetMap Premium extension (40).

### 2.3 Two-step floating catchment area methodology

In the absence of specific data on the supply-seeking behavior of individuals, we may assume that such behaviors depend on travel distance or time. We can then employ distance decay functions to reflect that individuals may favor geographically proximate supplies (9, 18, 41). In spatial accessibility studies, various distance decay functions have been used, including step-based and continuous decay (23, 41, 42). Continuous decay functions include the Gaussian (13, 21, 27, 43), log-logistic (44, 45), and kernel density (10, 18, 22). The distance decay function requires the specification of the maximum distance or maximum travel time that individuals are willing to undertake (hereafter referred to as the catchment area). Previous studies have utilized a variety of catchment areas for travel via private car, including 30 min (9, 15, 20, 21, 44), 45 min (46), 60 min (45, 47), and 90 or more minutes (18, 27, 45). Gu et al. (46) support the 30-min travel time as a standard threshold for healthcare access, but suggest that 60 min may be more appropriate for regional-level accessibility. Conversely, other literature argues that the 30-min threshold is, at best, arbitrarily defined (12). The catchment area can also be defined as the travel time in which a certain percentage of the population of interest has access to the service of interest (43). Comprehensive assessments and comparison of distance decay functions can be found in other works (23, 41, 42), such as Chen and Jia (23), who performed a comparative analysis of both distance decay functions and the selected maximum catchment area (*d*_max_).

For our analysis, we selected two kernel density distance decay functions both of which have been previously used to examine accessibility with FCA methods. The first is the Epanechnikov function proposed by Dai and Wang (18), which is defined as


$$f\left(d_{ji},d_{\max}\right)=\;\{\begin{array}{cc} \frac{3}{4}\left[1-{\left(\frac{d_{ji}}{d_{\max}}\right)}^{2}\right], & if\;d_{ji}\;\leq \;d_{\max}\\ 0, & if\;d_{ji}\;>\;d_{\max} \end{array}$$

In this function, *d*_*ji*_ represents the travel time between supply location *j* (e.g. CPAN clinic) and demand location *i* (e.g., county centroid), and *d*_max_ is the maximum time individuals are willing to travel (the catchment area). A later study by Polzin et al. (10) proposed the Quartic function, defined as


$$f\left(d_{ji},d_{init},\;d_{\max}\right)=\;\{\begin{array}{cc} 1, & if\;0\;<\;d_{ji}\;<\;d_{init}\\ \frac{15}{16}\left[1-{\left(\frac{d_{ji}}{d_{\max}}\right)}^{2}\right], & if\;d_{init}\;\leq \;d_{ji}\;\leq \;d_{\max}\\ 0, & if\;d_{ji}\;>\;d_{\max} \end{array}$$

where *d*_*init*_ represents the initial range of travel times that do not pose any travel impedance and is selected by the investigator (e.g., 15 min). Compared to the Epanechnikov function, the Quartic function imposes a more lenient penalty for shorter travel times and a more stringent penalty for longer ones, partially by including the *d*_*init*_.

Next, in the first step, the 2SFCA calculates the supply-to-demand ratio *R*_*j*_ for each supply measure *S*_*j*_ at supply point *j* and the weighted demand measure *P*_*i*_ for demand point *i* as below:


$$\begin{array}{l} R_{j}=\;\frac{S_{j}}{\sum_{i\;\in \;\left\{d_{ji}\;\leq \;d_{\max}\right\}}P_{i}\cdot f\left(d_{ji},\;d_{\max}\right)} \end{array}$$

Here, the supply measure *S*_*j*_ could be numeric metrics such as the number of hospital beds, the amount of available product, or the number of services or care providers available. If there is no information on supply capacity, then *S*_*j*_ may be set equal to 1 to represent the supply's presence. For our analysis, *S*_*j*_ is set equal to 1. We considered the estimated county-level ADHD counts (*P*_*i*_), which was calculated as the product of the estimated county-level prevalence and the corresponding county-level pediatric population size.

In the second step, the supply-to-demand ratios *R*_*j*_ are combined in a weighted sum over each demand point to create the Spatial Accessibility Index (SPAI):


$$\begin{array}{l} SPAI_{i}=\;\underset{j\;\in \;\left\{d_{ji}\;\leq \;d_{\max}\right\}}{\sum }R_{j}\;\cdot f\left(d_{ji},\;d_{\max}\right) \end{array}$$

In the absence of data on people's health-seeking travel behavior, the reliance upon often arbitrarily determined travel thresholds or travel impedance may be problematic as the SPAI may vary substantially across selected values. The Spatial Access Ratio (SPAR) was proposed to minimize some of the uncertainty introduced by impedance parameter selections (48). The SPAR is calculated by dividing the SPAI of each demand point by the average SPAI value of all demand points. SPAR values of less than 1 indicate lower-than-average access and values greater than 1 indicate higher-than-average access.

All data processing, statistical analyses, and comparisons were performed in R (version 4.3.0) (49).

## 3 Results

**Figure 2:**
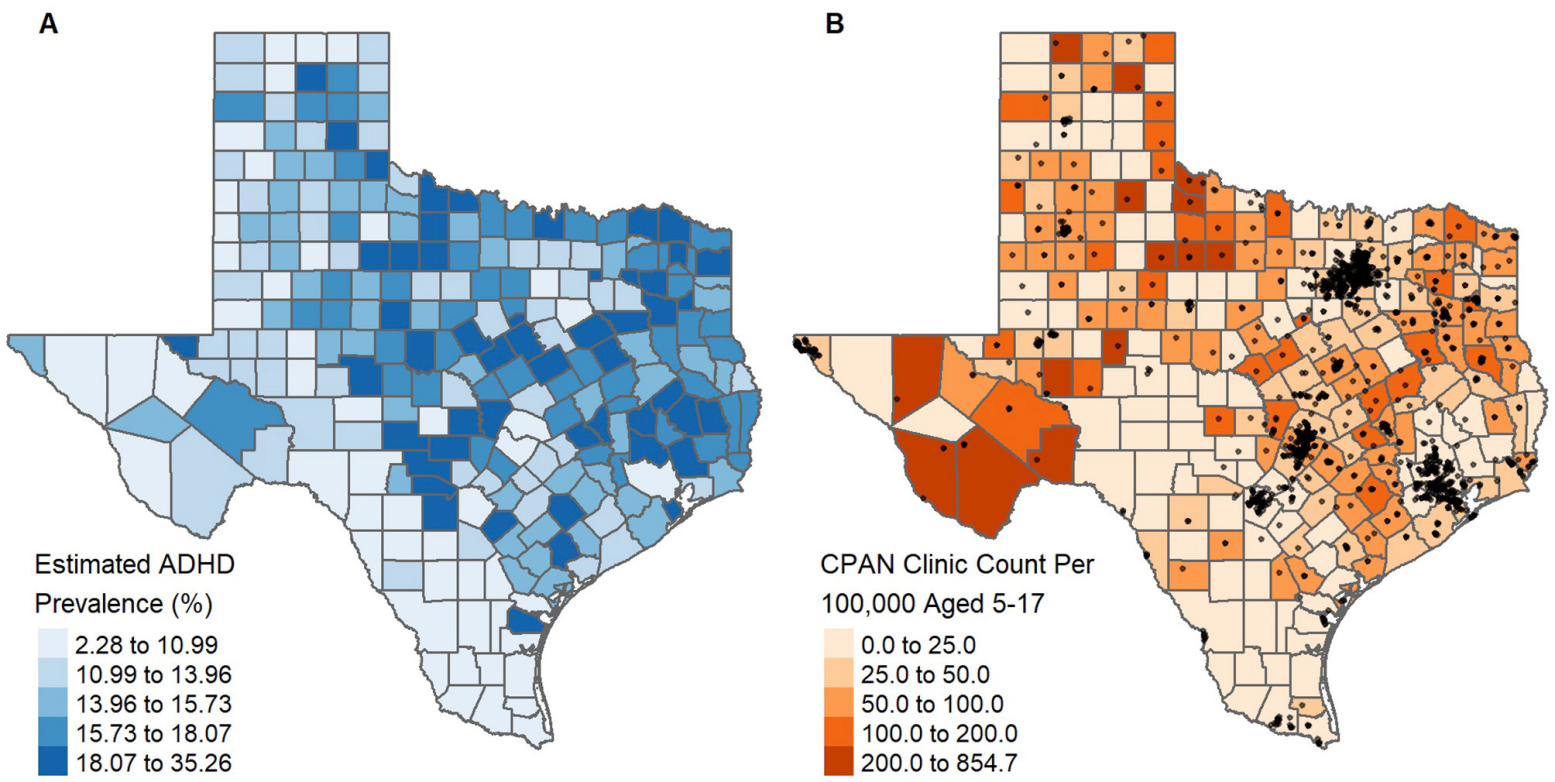
Estimated county-level ADHD prevalence among children and adolescents aged 5–17, as obtained by Zgodic et al. (37) **(A)** and the number of clinics enrolled in a statewide program per 100,000 aged 5–17 with clinic locations (black dots) overlaid **(B)**.

We applied the 2SFCA method with a continuous kernel density distance decay function to the Texas clinic dataset described above and ADHD prevalence data provided by Zgodic et al. (37). The estimated county-level ADHD prevalence among children and adolescents aged 5–17 presented substantial geographical variation, ranging from 2.28% in Kenedy County to 35.26% in Galveston County (median [IQR]: 14.94 [11.93, 17.40]) (Figure 2A). The estimated ADHD count, reflecting pediatric demand, was highly skewed (median [IQR]: 476.5 [143, 1485]) with urban areas such as Houston, Dallas, Austin, and San Antonio showing higher counts compared to rural areas.

At the time of analysis, clinics enrolled in the 1st year of the program were present in 193 of the 254 Texas counties. However, the number of enrolled clinics per county varied substantially and was highly skewed with 73.06% (141 of 193) of counties having 1–5 enrolled clinics, while 2.59% (5 of 193) had over 100 (Supplementary Figure 1C). Moreover, urban counties had more clinics compared to rural counties (Urban: 6 [1, 18.75]; Rural: 1 [0, 2]), with clinics heavily clustered around urban centers (Supplementary Figure 2). We also calculated the number of enrolled clinics per 100,000 population aged 5–17 at the county level, which ranged from 0 to 854.7 (median [IQR]: 40.48 [9.69, 74.90]) (Figure 2B). Accounting for the study population size, rural counties tended to have a higher, but more variable, enrolled clinic count per 100,000 (Urban: 29.09 [11.56, 54.01]; Rural: 47.62 [0, 85.21]).

**Figure 3:**
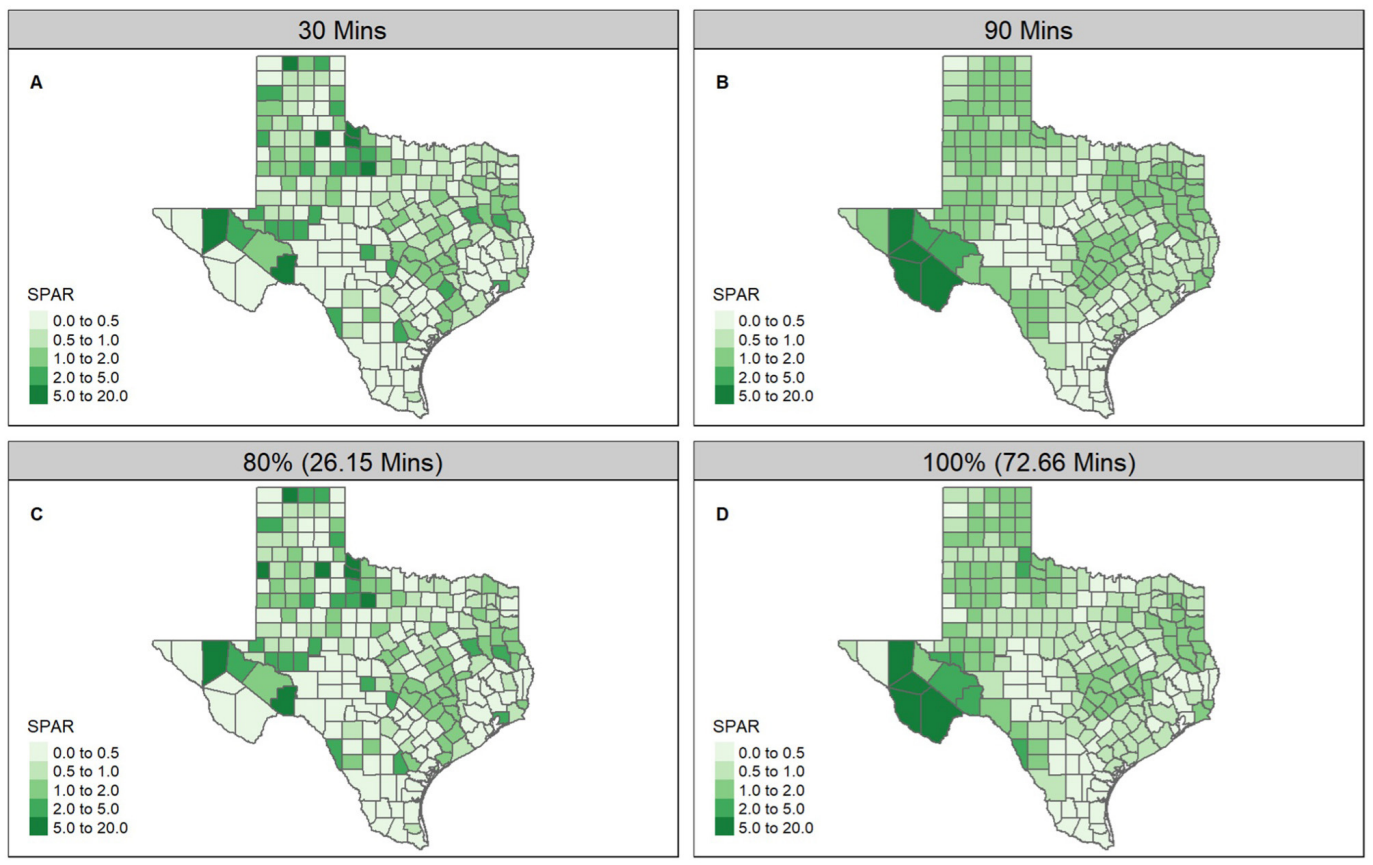
Selected SPAR calculated using individual enrolled clinic-county pairs, and the Epanechnikov distance decay function with differing catchment selections: **(A)** 30-min travel time, **(B)** 90-min travel time, **(C)** 80% of the population with access, and **(D)** 100% of the population with access.

For the kernel density 2SFCA, we compared accessibility across two kernel distance decay functions, the Epanechnikov and Quartic, and across a selection of maximum catchment areas (*d*_max_) based on travel time thresholds (30, 45, 60, 90, and 120 min) and thresholds determined by the percent of the population with access to at least one point of supply (80%, 85%, 90%, 95%, and 100%). SPAR values, representing spatial accessibility scores, ranged from 0 to 15.88 (SD: 1.62) for a 30-min travel time catchment. As travel time increased to 90 min, the geographic spread and variability of SPAR reduced to a range of 0.022–11.20 (SD: 0.98) (Figure 3). Urban counties had lower average SPAR values (30 min: 0.48 [0.25, 0.79]; 90 min: 0.89 [0.63, 1.20]) compared to rural counties at the same catchment criteria (30 min: 0.70 [0.20, 1.45]; 90 min: 0.83 [0.50, 1.21]), though this difference was less pronounced at larger catchments. Between the 30- and 90-min catchments, specific regions in West Texas, including Jeff Davis, Presidio, and Brewster counties, exhibited the greatest change in SPAR, transitioning from the very low accessibility category (SPAR values between 0.0 and 0.5) at 30 min to the highest accessibility (SPAR values above 5) at 90 min (Figure 3). In contrast, portions of central and southern Texas consistently fell within the lowest accessibility category across all specified catchments and distance decay functions (Supplementary Figures 3–6). Highly populated counties such as Harris (including Houston), Dallas, and Tarrant (including Fort Worth) had an improvement regarding access category between 30- and 90-min (Figure 3), while Travis County, which includes Austin, remained within the same access category between both catchment selections. Changing the maximum catchment area by travel time resulted in distinct variations in the SPAR values across Texas, presenting noticeable geographic patterns. When holding the travel time constant but varying the decay functions in defining the catchment areas, SPAR values were highly correlated (Pearson's correlation coefficient r: 0.92–0.99) between the Epanechnikov and Quartic kernel distance decay functions (Supplementary Figures 7, 8 and Supplementary Table 1) Finally, achieving 80% access for the demand population required a catchment of 26.15 min, while 100% access required 72.66 min (Figure 3).

**Figure 4:**
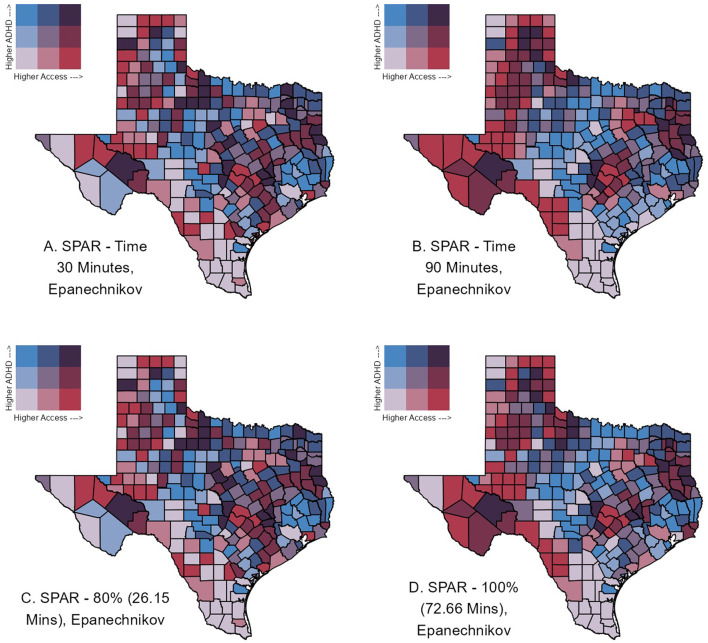
Bivariate county-level maps of Texas assessing estimated ADHD prevalence, as obtained by Zgodic et al. (37), and accessibility as measured by the Spatial Accessibility Ratio (SPAR) derived from the 2SFCA. ADHD prevalence was divided into terciles: low (0%−12.82%), moderate (12.82%−16.34%), and high (>16.34%). Similarly, SPAR was divided by terciles for each catchment area: low (Q1), medium (Q2), and high (Q3). Counties in blue shades (upper left tile) represent those with low access, but high demand and may be potential priority counties for future enrollment efforts.

**Figure 5:**
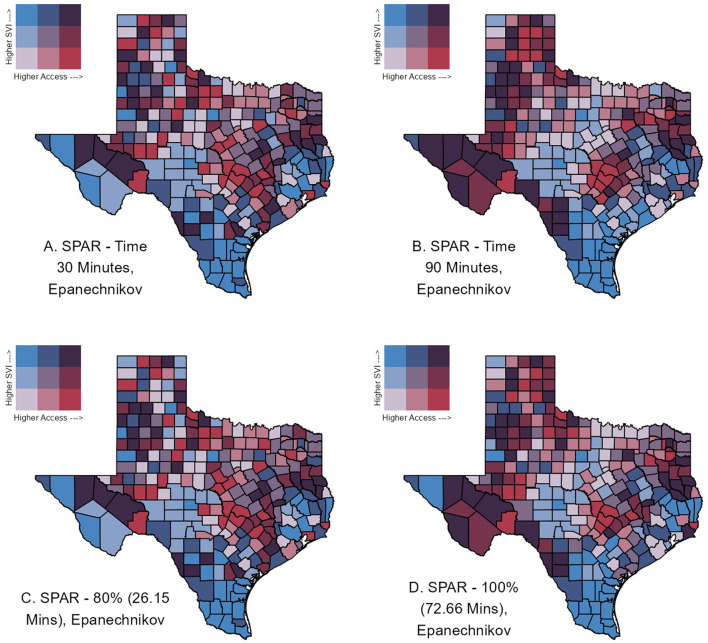
Bivariate county-level maps of Texas assessing the Social Vulnerability Index (SVI) and accessibility as measured by the Spatial Accessibility Ratio (SPAR) derived from the 2SFCA. SVI was divided into terciles: low (0–0.33), moderate (0.33–0.67), and high (>0.67). Similarly, SPAR was divided by terciles for each catchment area: low (Q1), medium (Q2), and high (Q3). Counties in blue shades (upper left tile) represent those with low access and high vulnerability.

Bivariate mapping is a useful approach to visually investigate the association of the SPAR with another relevant areal characteristic using a color scale to highlight areas potentially in need of new resource implementation. To illustrate this method, Figure 4 presents the bivariate map of estimated ADHD prevalence and county-level SPAR values for four selected catchments of 30-min or 90-min travel time, and 80% or 100% population with access. We categorized SPAR into terciles representing low, medium, and high access, for each of the four catchments. Estimated ADHD prevalence was also divided into equal-sized terciles of low, moderate, and high groups. Counties with high prevalence, but very low access, represent potential areas to prioritize for future program outreach and enrollment efforts. With a catchment of 30- and 90-min, 29 and 33 counties had high prevalence but very low access to enrolled clinics, respectively. Additionally, Figure 5 presents the bivariate map of county-level SVI and SPAR values for the same selected catchments. SVI was categorized into terciles, representing low, medium, and high vulnerability. Counties with low access, but high vulnerability may also be suitable areas to prioritize for future program allocation. For both 30- and 90-min catchments, 27 counties had high vulnerability, but very low access to enrolled clinics. Among these counties, several urban and highly vulnerable areas were identified, including Nueces, Polk, and Trinity counties, suggesting these counties may be suitable areas for future priority enrollment efforts.

## 4 Discussion

In this study, we compared the relative impact of key 2SFCA method inputs, specifically the distance decay function and catchment area. We then assessed the resulting spatial accessibility measures for clinics enrolled in a statewide child and adolescent psychiatric consultation program, specifically noting regions in Texas that consistently had poor access across method inputs. Pockets of low access were observed in central and southern Texas paired with high vulnerability. Further, a more spatially granular investigation of large urban centers will be required for a complete picture of access to pediatric mental health services. While we use this Texas clinic data, this study is not intended as an evaluation of the program, but as guidance for selecting spatial methods to evaluate future program enrollment or other intervention efforts.

The inclusion of distance decay functions in spatial accessibility analysis has been explored to account for travel impedance within the catchment. We considered the Epanechnikov and Quartic decay functions previously used to assess spatial accessibility (10, 18), and found that county-level SPAR were similar and highly correlated between both distance functions for the same catchment area value (Supplementary Table 1). This consistency aligns with previous studies (23, 41), suggesting that the specific choice of distance decay may be less critical in practice. Conversely, the selection of the catchment area exhibited a more significant impact on accessibility trends, as evidenced by the notable increase in SPAR values in counties like Jeff Davis, Presidio, and Brewster when the catchment was increased from 30- to 90-min. Smaller catchments (e.g., 30 min) displayed more discrete and local geographic variability, while larger catchments (e.g., 90 min) produced geographically smoother patterns of SPAR, as expected (see Figure 3). However, despite changes in the state-wide accessibility patterns, clusters of counties in central and southern Texas consistently remained in the lowest SPAR category, indicating low accessibility across all catchments.

While some general benchmarks exist in the FCA literature, catchment area specification is often arbitrary (12) and loosely grounded in empirical data on care-seeking behavior (12, 45, 46). Certain population characteristics have been previously investigated in relation to care-seeking behavior. Previous work has shown that residents of rural counties are often more willing to travel further for care than their urban counterparts (50, 51). Further, car ownership is not universal and urban residents in particular may utilize alternative modes of transportation, such as public transport, cycling, or walking (11, 13, 52, 53). Various catchments and multi-modal transportation have been proposed in the 2SFCA approach to address differences in supply capacity and care-seeking behavior. For example, Tao et al. (20) used facility size to inform supply catchments and Bauer and Groneberg (45) integrated different distance decay and catchments, to capture consumer behavior variations.

However, determining the optimal catchment area to accurately represent the “true” accessibility pattern remains a challenge since care-seeking behavior is often tied to specific healthcare needs of the study population and region. Data on specific care-seeking behavior to define the optimal catchment is often not available. For example, one study compared the realized and acceptable access to primary and specialist care (54). Various demographic factors influenced acceptable differences including age and town size for primary care and income for specialist care. To the best of our knowledge, no such study exists for Texas; factors influencing realized and acceptable access to primary and specialist services are likely to differ. Other dimensions of accessibility, notably affordability (e.g., out-of-pocket costs, insurance coverage), are also likely to influence realized access, but are not incorporated into the FCA framework. Programs like CPAN may help to overcome other accessibility barriers by linking primary care providers with specialist consultations, which may directly improve patient care and help to build PCP capacity to manage future similar cases (55).

Comparing the resulting SPAR values to estimated ADHD prevalence patterns (Figure 4) and SVI (Figure 5) revealed a more nuanced pattern across counties. Assessing SPAR alone, central and southern Texas counties fell within the lowest accessibility category. Priority counties may further be specified by identifying which counties have particularly low access and/or high social vulnerability, but relatively higher demand. Most counties in southern Texas had low access coupled with low demand. By contrast, counties such as Bandera, Kerr, Kimble, and Neuces may be higher priority counties for future CPAN enrollment as they exhibit very low access but fall within the highest tercile of ADHD prevalence across multiple applied catchment areas. However, the observed bivariate patterns may be misleading, particularly in large urban areas (e.g., Harris County), as we were unable to account for clinic capacity. Further, consideration for varying levels of ADHD diagnosis could have affected the findings. A complete discussion on patterns of diagnosis for ADHD is beyond the scope of this paper, as ADHD prevalence was one example measure selected among various other mental health conditions that affect children and adolescents.

Our study has a few limitations of note. First, we were unable to incorporate a measure of supply capacity (e.g., the number of primary care physicians, pediatric patient load) due to data limitations and restrictions, whereby only the location of the clinics was available. This may have had the strongest impact on urban clinics, which may be equipped to handle a larger influx of patients than their rural counterparts. The lack of supply capacity may explain why some urban areas like Houston, where the Texas Medical Center is located, received lower accessibility scores. Second, the 2SFCA method provides a simplification of travel behavior complexity. Improving data availability on care-seeking behavior, including the relative importance of distance impedance versus other barriers to care such as out-of-pocket costs, is crucial to inform the catchment parameters of the FCA methodology and better guide policy. Travel distance has been shown to reflect a significant barrier to care, especially for rural regions (51, 56); however, with the rise of Telemedicine as a popular alternative for seeking health services (particularly in mental health services), the role of distance as a barrier to care may shift, necessitating a reevaluation of FCA and other accessibility approaches. Finally, our analysis only provides a broad, state-wide assessment of accessibility. The 2SFCA approach assumes a uniform population distribution within the catchment area, which may not reflect the actual population distribution, particularly for catchment areas that include large urban areas and small rural counties. The realized accessibility of more granular units, such as neighborhoods, may be obscured by county-level patterns.

### 4.1 Conclusions and future directions

FCA methods are useful tools to assess healthcare resource accessibility, though its application can further be improved by greater data availability on care-seeking behavior and supply capacity. Since the original 2SFCA, substantial work has been made to address identified shortcomings through various modifications and adaptations. These include the Three-Step Floating Catchment Area (3SFCA) approach that incorporated a measure of supply competition through the addition of the Huff model (57), and inclusion of the Huff model alongside various distance decay and catchment sizes into the integrated FCA (iFCA) (45). Other literature has explored the implementation of multiple modes of transportation into the FCA approach (11, 13, 52, 53, 58). Future work could consider applying other FCA methods to better control for supply competition and variable catchments based on demographic preferences and willingness to travel. Additionally, future work in large urban centers should further investigate accessibility by integrating clinic capacity data and exploring multi-modal transportation options to refine accessibility estimates in these areas. Counties in southern and central Texas are priority candidates to expand enrollment in the CPAN program. Future work should further investigate the accessibility to pediatric mental health services in large urban centers to better guide public health policy and resource allocation in those areas.

## Data Availability

Publicly available datasets were analyzed in this study. This data can be found at: Data on CPAN clinics is internal to the CPAN program and is not publicly available. The data on ADHD prevalence by Zgodic et al. (37) can be found as Supplementary material to their manuscript: https://www.sciencedirect.com/science/article/pii/S1047279723000066#sec0011. SVI (2020) data can be found at: https://www.atsdr.cdc.gov/place-health/php/svi/svi-data-documentation-download.html. RUCC (2013) data can be found at: https://www.ers.usda.gov/data-products/rural-urban-continuum-codes.

## References

[1] Gunja MZ Gumas ED Williams II RD. U.S. Health Care from a Global Perspective, 2022: Accelerating Spending, Worsening Outcomes. New York City, NY: Commonwealth Fund (2023).

[2] Schneider EC Shah A Doty MM Tikkanen R Fields K Williams RDI. Mirror, Mirror 2021 – Reflecting Poorly: Health Care in the U.S. Compared to Other High-Income Countries. New York City, NY: Commonwealth Fund (2021).

[3] Penchansky R Thomas JW. The concept of access: definition and relationship to consumer satisfaction. Med Care. (1981) 19:127. 10.1097/00005650-198102000-00001 7206846

[4] Radke J Mu L. Spatial decompositions, modeling and mapping service regions to predict access to social programs. Geograph Inform Sci. (2000) 6:105–12. 10.1080/10824000009480538

[5] Shen Q. Location characteristics of inner-city neighborhoods and employment accessibility of low-wage workers. Environ Plann B Plann Des. (1998) 25:345–65. 10.1068/b250345

[6] Weibull JW. An axiomatic approach to the measurement of accessibility. Reg Sci Urban Econ. (1976) 6:357–79. 10.1016/0034-3331(76)90002-6

[7] Luo W Wang F. Measures of spatial accessibility to health care in a GIS environment: synthesis and a case study in the Chicago Region. Environ Plann B: Plann Design. (2003) 30:865–84. 10.1068/b29120 34188345 PMC8238135

[8] Luo W Wang F. Spatial accessibility to primary care and physician shortage area designation: a case study in Illinois with GIS approaches. Geograph Inform Syst Health Appl. (2003) 2003:260–78. 10.4018/978-1-59140-042-4.ch015

[9] Luo W Qi Y. An enhanced two-step floating catchment area (E2SFCA) method for measuring spatial accessibility to primary care physicians. Health Place. (2009) 15:1100–7. 10.1016/j.healthplace.2009.06.002 19576837

[10] Polzin P Borges J Coelho A. An extended kernel density two-step floating catchment area method to analyze access to health care. Environ Plan B: Plan Design. (2014) 41:717–35. 10.1068/b120050p

[11] Mao L Nekorchuk D. Measuring spatial accessibility to healthcare for populations with multiple transportation modes. Health Place. (2013) 24:115–22. 10.1016/j.healthplace.2013.08.008 24077335

[12] Jamtsho S Corner R Dewan A. Spatio-temporal analysis of spatial accessibility to primary health care in Bhutan. ISPRS Int J Geo-Inform. (2015) 4:1584–604. 10.3390/ijgi4031584

[13] Zhou X Yu Z Yuan L Wang L Wu C. Measuring accessibility of healthcare facilities for populations with multiple transportation modes considering residential transportation mode choice. ISPRS Int J Geo-Inform. (2020) 9:394. 10.3390/ijgi9060394

[14] Liu L Lyu H Zhao Y Zhou D. An improved two-step floating catchment area (2SFCA) method for measuring spatial accessibility to elderly care facilities in Xi'an, China. Int J Environ Res Public Health. (2022) 19:11465. 10.3390/ijerph191811465 36141737 PMC9517364

[15] Rauch S Stangl S Haas T Rauh J Heuschmann PU. Spatial inequalities in preventive breast cancer care: a comparison of different accessibility approaches for prevention facilities in Bavaria, Germany. J Transport Health. (2023) 29:101567. 10.1016/j.jth.2023.101567

[16] Zhou Y Beyer KMM Laud PW. An adapted two-step floating catchment area method accounting for urban–rural differences in spatial access to pharmacies. J Pharm Health Serv Res. (2021) 12:69–77. 10.1093/jphsr/rmaa022 33717229 PMC7938828

[17] Li Z Liang Z Feng L Fan Z. Beyond accessibility: a multidimensional evaluation of urban park equity in Yangzhou, China. ISPRS Int J Geo-Inform. (2022) 11:429. 10.3390/ijgi11080429

[18] Dai D Wang F. Geographic disparities in accessibility to food stores in southwest Mississippi. Environ Plan B: Plan Design. (2011) 38:659–77. 10.1068/b36149

[19] Raeesi A Kiani B Hesami A. Access to the COVID-19 services during the pandemic - a scoping review. Geospatial Health. (2022) 17:1079. 10.4081/gh.2022.1079 35352541

[20] Tao R Downs J Beckie TM Chen Y McNelley W. Examining spatial accessibility to COVID-19 testing sites in Florida. Ann GIS. (2020) 26:319–27. 10.1080/19475683.2020.1833365

[21] Ghorbanzadeh M Kim K Erman Ozguven E Horner MW. Spatial accessibility assessment of COVID-19 patients to healthcare facilities: a case study of Florida. Travel Behav Soc. (2021) 24:95–101. 10.1016/j.tbs.2021.03.004 33777697 PMC7980178

[22] Shen X. Accessibility calculation and equality evaluation of medical facilities for COVID-19 pandemic treatment: a case study of the Wuhan metropolitan development zone. PLoS ONE. (2022) 17:e0272458. 10.1371/journal.pone.0272458 35917292 PMC9345339

[23] Chen X Jia P A. Comparative analysis of accessibility measures by the two-step floating catchment area (2SFCA) method. Int J Geograph Inform Sci. (2019) 33:1739–58. 10.1080/13658816.2019.1591415

[24] Wang S Wang M Liu Y. Access to urban parks: comparing spatial accessibility measures using three GIS-based approaches. Comput Environ Urban Syst. (2021) 90:101713. 10.1016/j.compenvurbsys.2021.101713

[25] Luan H Li G Duncan DT Sullivan PS Ransome Y. Spatial accessibility of pre-exposure prophylaxis (PrEP): different measure choices and the implications for detecting shortage areas and examining its association with social determinants of health. Ann Epidemiol. (2023) 86:72–9.e3. 10.1016/j.annepidem.2023.07.004 37453464

[26] TCMHCC – Texas Child Mental Health Care Consortium. (2023). Available at: https://tcmhcc.utsystem.edu/ (accessed 12 July 2023).

[27] Chen L Chen T Lan T Chen C Pan J. The contributions of population distribution, healthcare resourcing, and transportation infrastructure to spatial accessibility of health care. Inquiry. (2023) 60:00469580221146041. 10.1177/00469580221146041 36629371 PMC9837279

[28] Dickson C Ramsay J VandeBurgh J. Barriers for ethnic minorities and low socioeconomic status pediatric patients for behavioral health services and benefits of an integrated behavioral health model. Pediatr Clin North Am. (2021) 68:651–8. 10.1016/j.pcl.2021.02.013 34044991

[29] Olatunji G Faturoti O Jaiyeoba B Toluwabori AV Adefusi T Olaniyi P. Navigating unique challenges and advancing equitable care for children with ADHD in Africa: a review. Ann Med Surg (Lond). (2023) 85:4939–46. 10.1097/MS9.0000000000001179 37811061 PMC10553014

[30] Amos A Coleman M Spring Walsh B Gardiner FW. Remoteness and socioeconomic status reduce access to specialist mental health care across Australia. Austr Psychiatry. (2023) 31:19–26. 10.1177/10398562221139129 36378120

[31] Cummings JR Allen L Clennon J Ji X Druss BG. Geographic access to specialty mental health care across high- and low-income US communities. JAMA Psychiatry. (2017) 74:476–84. 10.1001/jamapsychiatry.2017.0303 28384733 PMC5693377

[32] Kolko DJ Perrin E. The integration of behavioral health interventions in children's health care: services, science, and suggestions. J Clin Child Adolesc Psychol. (2014) 43:216–28. 10.1080/15374416.2013.862804 24588366 PMC4011180

[33] Walter HJ Vernacchio L Correa ET. Five-phase replication of behavioral health integration in pediatric primary care. Pediatrics. (2021) 148:e2020001073. 10.1542/peds.2020-001073 34210739

[34] Geocoding API overview. Google for Developers. (2023). Available at: https://developers.google.com/maps/documentation/geocoding/overview (accessed 12 July 2023).

[35] U.S. Census Bureau. 2017-2021 American Community Survey 5-year Public Use Microdata samples. Retrieved from Census API. (2023).

[36] Bureau UC. Centers of Population. Census.gov. (2023). Available at: https://www.census.gov/geographies/reference-files/time-series/geo/centers-population.html (accessed 12 July 2023.)

[37] Zgodic A McLain AC Eberth JM Federico A Bradshaw J Flory K. County-level prevalence estimates of ADHD in children in the United States. Ann Epidemiol. (2023) 79:56–64. 10.1016/j.annepidem.2023.01.006 36657694 PMC10099151

[38] USDA ERS. Rural-Urban Continuum Codes. Available at: https://www.ers.usda.gov/data-products/rural-urban-continuum-codes.aspx (accessed 13 July 2023).

[39] Flanagan BE Gregory EW Hallisey EJ Heitgerd JL Lewis B. A social vulnerability index for disaster management. J Homeland Sec Emerg Manage. (2011) 8:1792. 10.2202/1547-7355.1792

[40] ArcGIS [GIS software]. Version 10.8. Redlands, CA: Environmental Systems Research Institute, Inc. (2010).

[41] McGrail MR. Spatial accessibility of primary health care utilising the two step floating catchment area method: an assessment of recent improvements. Int J Health Geogr. (2012) 11:50. 10.1186/1476-072X-11-50 23153335 PMC3520708

[42] Wang F. Measurement, optimization, and impact of health care accessibility: a methodological review. Ann Assoc Am Geogr. (2012) 102:1104–12. 10.1080/00045608.2012.657146 23335813 PMC3547595

[43] Jin T Cheng L Wang K Cao J Huang H Witlox F. Examining equity in accessibility to multi-tier healthcare services across different income households using estimated travel time. Transport Policy. (2022) 121:1–13. 10.1016/j.tranpol.2022.03.014

[44] Fowler D Middleton P Lim S. Extending floating catchment area methods to estimate future hospital bed capacity requirements. Spat Spatiotemporal Epidemiol. (2022) 43:100544. 10.1016/j.sste.2022.100544 36460455

[45] Bauer J Groneberg DA. Measuring spatial accessibility of health care providers – introduction of a variable distance decay function within the floating catchment area (FCA) method. PLoS ONE. (2016) 11:e0159148. 10.1371/journal.pone.0159148 27391649 PMC4938577

[46] Gu Z Luo X Tang M Liu X. Does the edge effect impact the healthcare equity? An examination of the equity in hospitals accessibility in the edge city in multi-scale. J Transport Geography. (2023) 106:103513. 10.1016/j.jtrangeo.2022.103513

[47] Matthews KA Gaglioti AH Holt JB Wheaton AG Croft JB. Estimating health service utilization potential using the supply-concentric demand-accumulation spatial availability index: a pulmonary rehabilitation case study. Int J Health Geogr. (2020) 19:30. 10.1186/s12942-020-00224-2 32746848 PMC7397658

[48] Wan N Zhan FB Zou B Chow E. A relative spatial access assessment approach for analyzing potential spatial access to colorectal cancer services in Texas. Appl Geograp. (2012) 32:291–9. 10.1016/j.apgeog.2011.05.001

[49] R Core Team. R: A Language and Environment for Statistical Computing. Vienna: R Foundation for Statistical Computing (2022). Available at: https://www.R-project.org/

[50] McGrail MR Humphreys JS Ward B. Accessing doctors at times of need–measuring the distance tolerance of rural residents for health-related travel. BMC Health Serv Res. (2015) 15:212. 10.1186/s12913-015-0880-6 26022391 PMC4446808

[51] Arcury TA Gesler WM Preisser JS Sherman J Spencer J Perin J. The effects of geography and spatial behavior on health care utilization among the residents of a rural region. Health Serv Res. (2005) 40:135–55. 10.1111/j.1475-6773.2005.00346.x 15663706 PMC1361130

[52] Langford M Higgs G Fry R. Multi-modal two-step floating catchment area analysis of primary health care accessibility. Health Place. (2016) 38:70–81. 10.1016/j.healthplace.2015.11.007 26798964

[53] Park J Goldberg DW. A review of recent spatial accessibility studies that benefitted from advanced geospatial information: multimodal transportation and spatiotemporal disaggregation. ISPRS Int J Geo-Inform. (2021) 10:532. 10.3390/ijgi10080532

[54] Weinhold I Wende D Schrey C Militzer-Horstmann C Schang L Sundmacher L. Assessing patients' acceptable and realised distances to determine accessibility standards for the size of catchment areas in outpatient care. Health Policy. (2022) 126:1180–6. 10.1016/j.healthpol.2022.08.011 36180282

[55] Bifulco L Grzejszczak L Velez I Angelocci T Anderson D A. qualitative investigation of uninsured patient and primary care provider perspectives on specialty care eConsults. BMC Health Serv Res. (2023) 23:1133. 10.1186/s12913-023-10086-6 37864170 PMC10589958

[56] Douthit N Kiv S Dwolatzky T Biswas S. Exposing some important barriers to health care access in the rural USA. Public Health. (2015) 129:611–20. 10.1016/j.puhe.2015.04.001 26025176

[57] Wan N Zou B Sternberg T. A 3-step floating catchment area method for analyzing spatial access to health services. Int J Geograph Inform Sci. (2012) 26:1073–89. 11.624987 10.1080/13658816.2011.624987

[58] Kaur Khakh A Fast V Shahid R. Spatial accessibility to primary healthcare services by multimodal means of travel: synthesis and case study in the City of Calgary. Int J Environ Res Public Health. (2019) 16:170. 10.3390/ijerph16020170 30634454 PMC6351935

