# Supplementary material for: A comparison of floating catchment area parameters with applications to a dataset of clinics enrolled in a statewide child and adolescent psychiatric consultation program
Source: Front Public Health. 2025 Feb 20;13:1498819. doi: 10.3389/fpubh.2025.1498819 (PMC11882419; doi:10.3389/fpubh.2025.1498819)
Supplement: Supplementary file 1 [file Supplementary_file_1.docx]

Supplementary Material

A Comparison of Floating Catchment Area Parameters with Applications to a Dataset of Clinics Enrolled in a Statewide Child and Adolescent Psychiatric Consultation Program

Jocelyn Hunyadi, MPH, Lara S. Savas, PhD, Kehe Zhang, MS, Jeanette E. Deason, MPH, Ryan Ramphul, PhD, Melissa F. Peskin, PhD, Erica L. Frost, MPH, Cici Bauer, PhD, MS

*** Correspondence:**
Cici Bauer, E819, 1200 Pressler St., Houston, TX 77030 USA
[cici.x.bauer@uth.tmc.edu](mailto:cici.x.bauer@uth.tmc.edu)

# Supplementary Figures and Tables


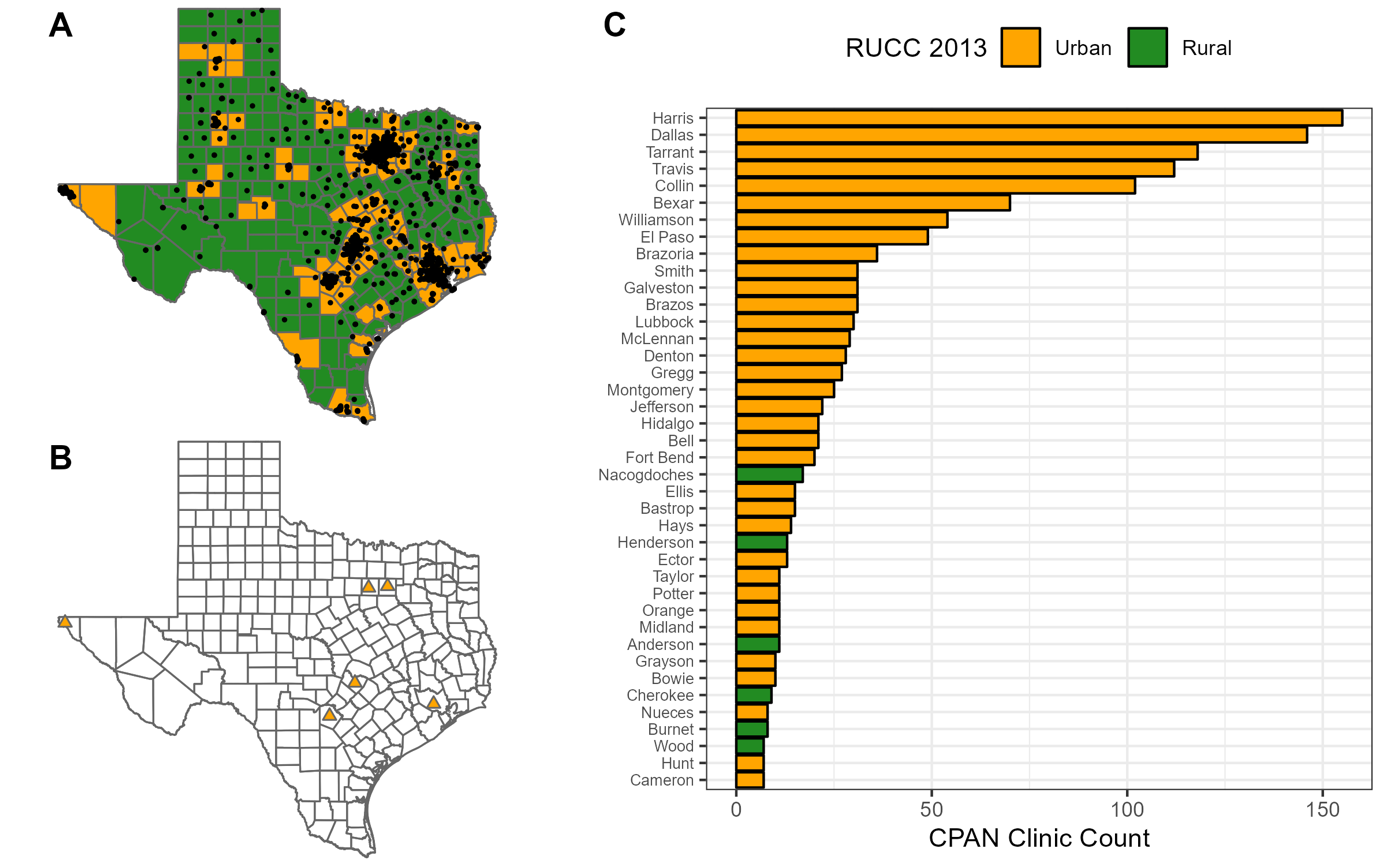


**Supplementary Figure 1**. CPAN clinics (A, black dots) are primarily located in counties with urban status (orange) and clustered around larger urban centers (B, orange triangles) in Texas: Houston, Dallas-Fort Worth, San Antonio, Austin, & El Paso. Clinic counts per county were heavily skewed with only 5 counties having over 100 clinics (C).


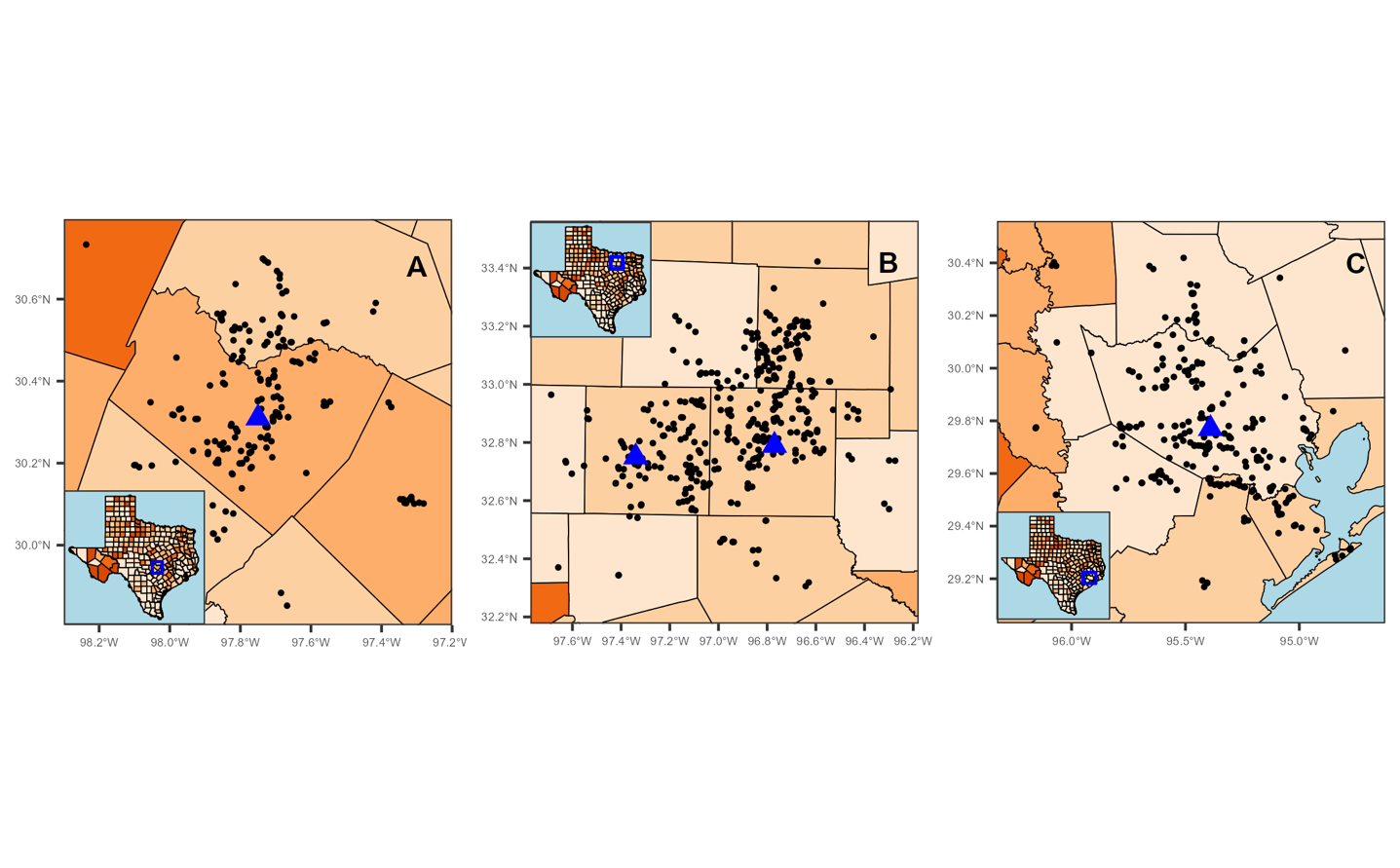


**Supplementary Figure 2**. A selection of inset maps of CPAN clinic count per 100,000 population aged 5-17 years. CPAN clinics (black dots) were primarily concentrated around large urban centers, including Austin (A), Dallas-Fort Worth (B), and Houston (C). The geographic centroid of each city’s location is denoted by the blue triangles.


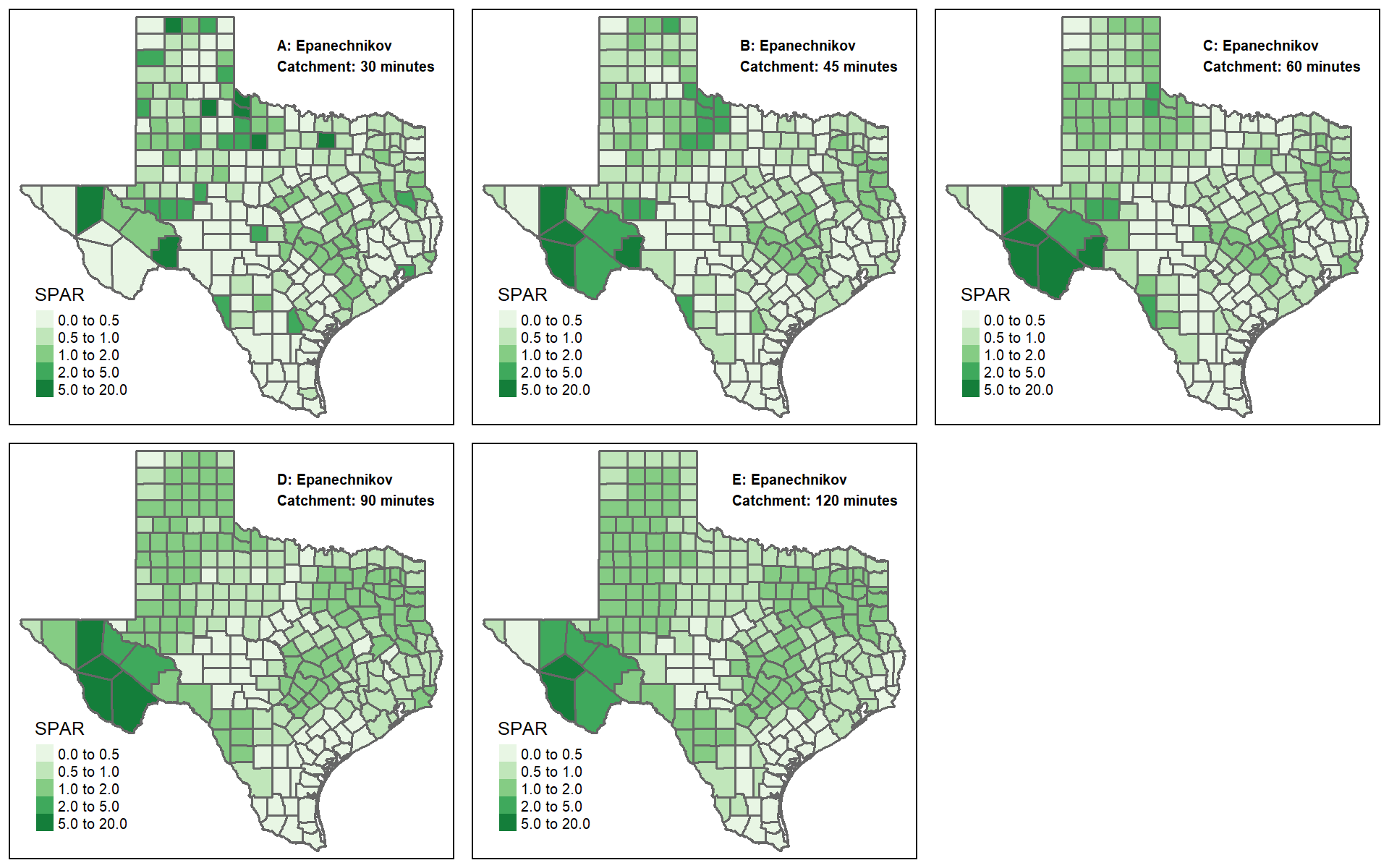


**Supplementary Figure 3**. County-level spatial accessibility ratio (SPAR) values for individual clinics using the Epanechnikov kernel density distance decay function across 5 catchment areas: 30 minutes (A), 45 minutes (B), 60 minutes (C), 90 minutes (D), and 120 minutes (E).


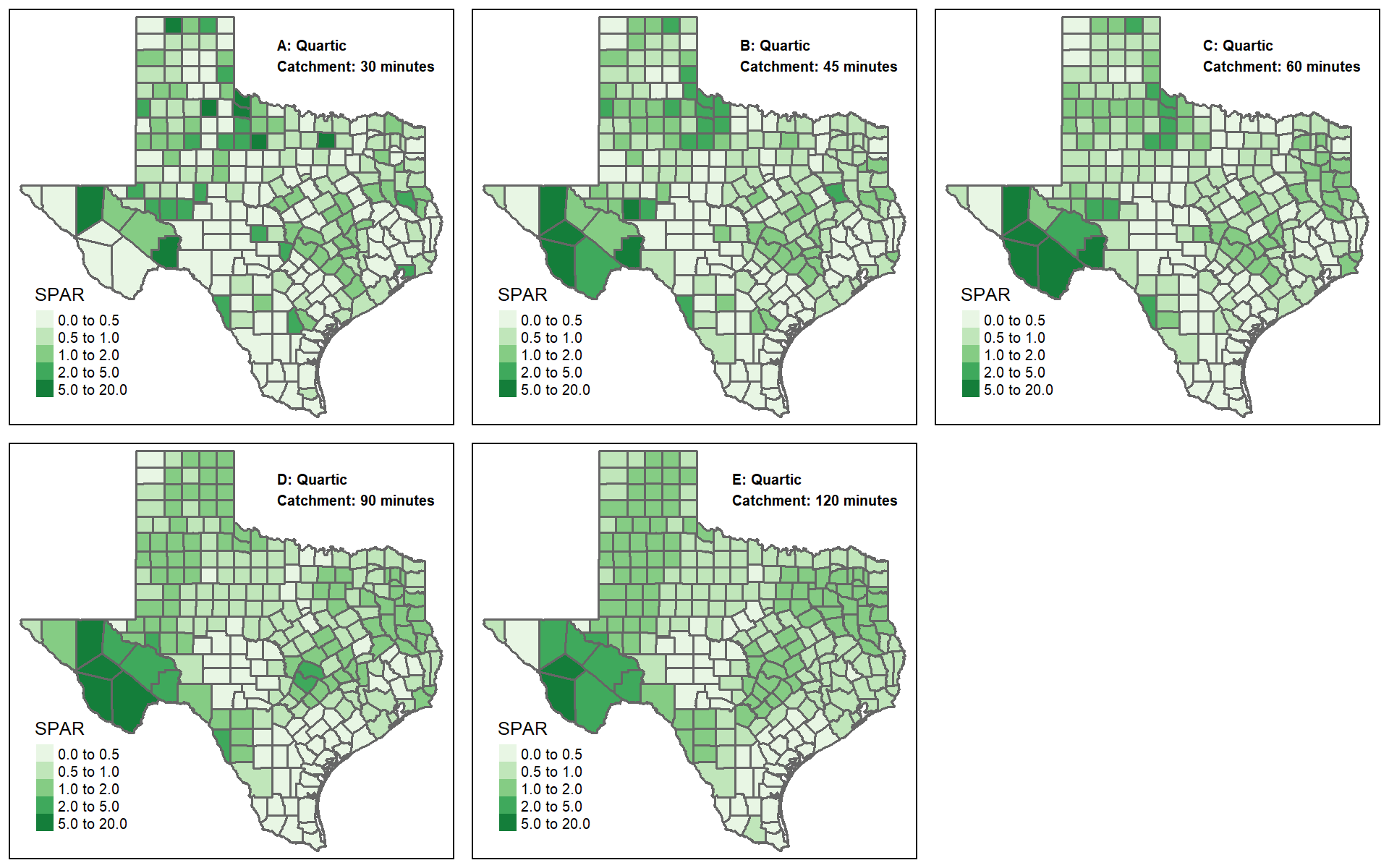


**Supplementary Figure 4**. County-level spatial accessibility ratio (SPAR) values for individual clinics using the Quartic kernel density distance decay function across 5 catchment areas: 30 minutes (A), 45 minutes (B), 60 minutes (C), 90 minutes (D), and 120 minutes (E).


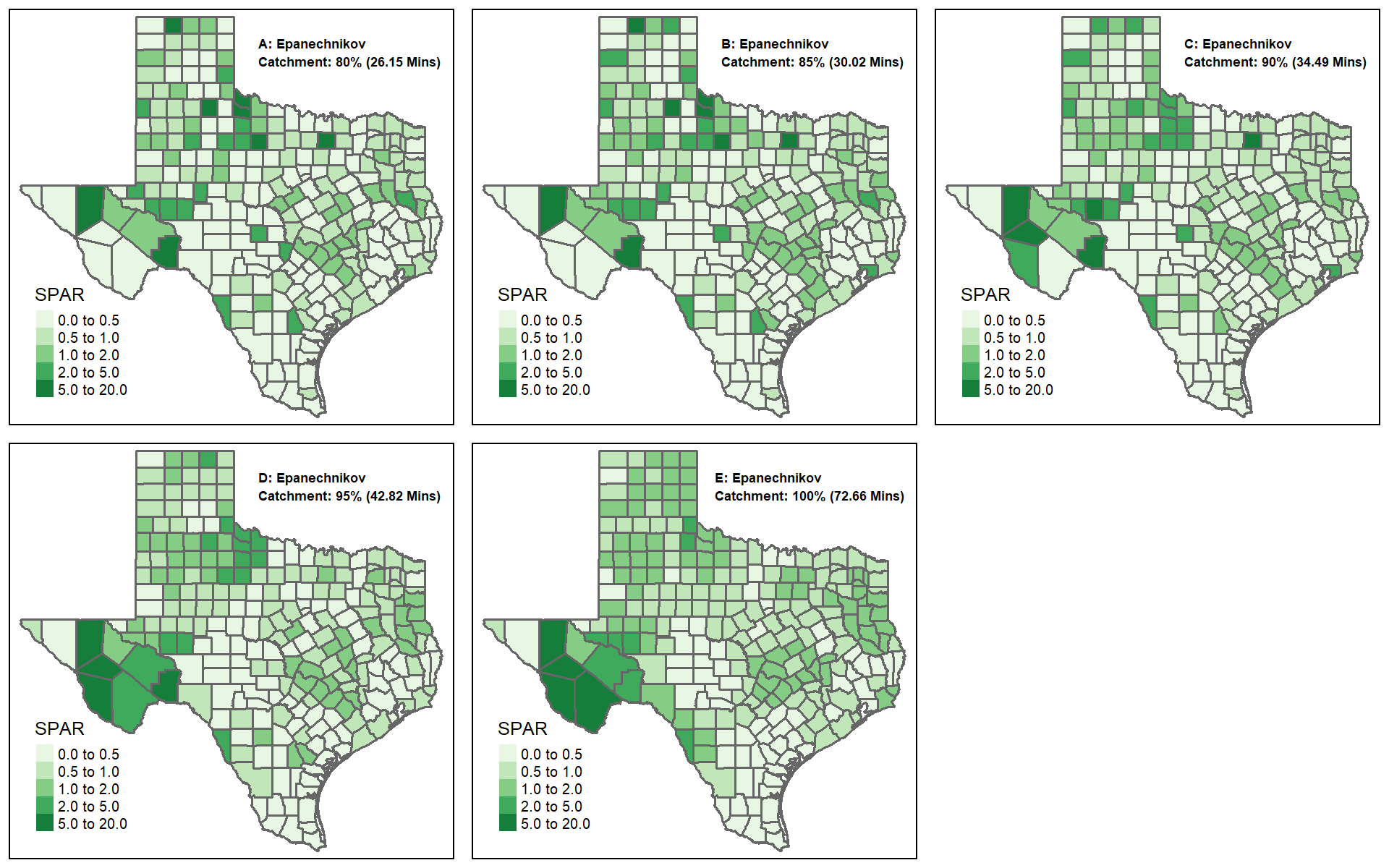


**Supplementary Figure 5**. County-level spatial accessibility ratio (SPAR) values for individual clinics using the Epanechnikov kernel density distance decay function across 5 catchment areas: 80% population access (A), 85% access (B), 90% access (C), 95% access (D), and 100% access (E).


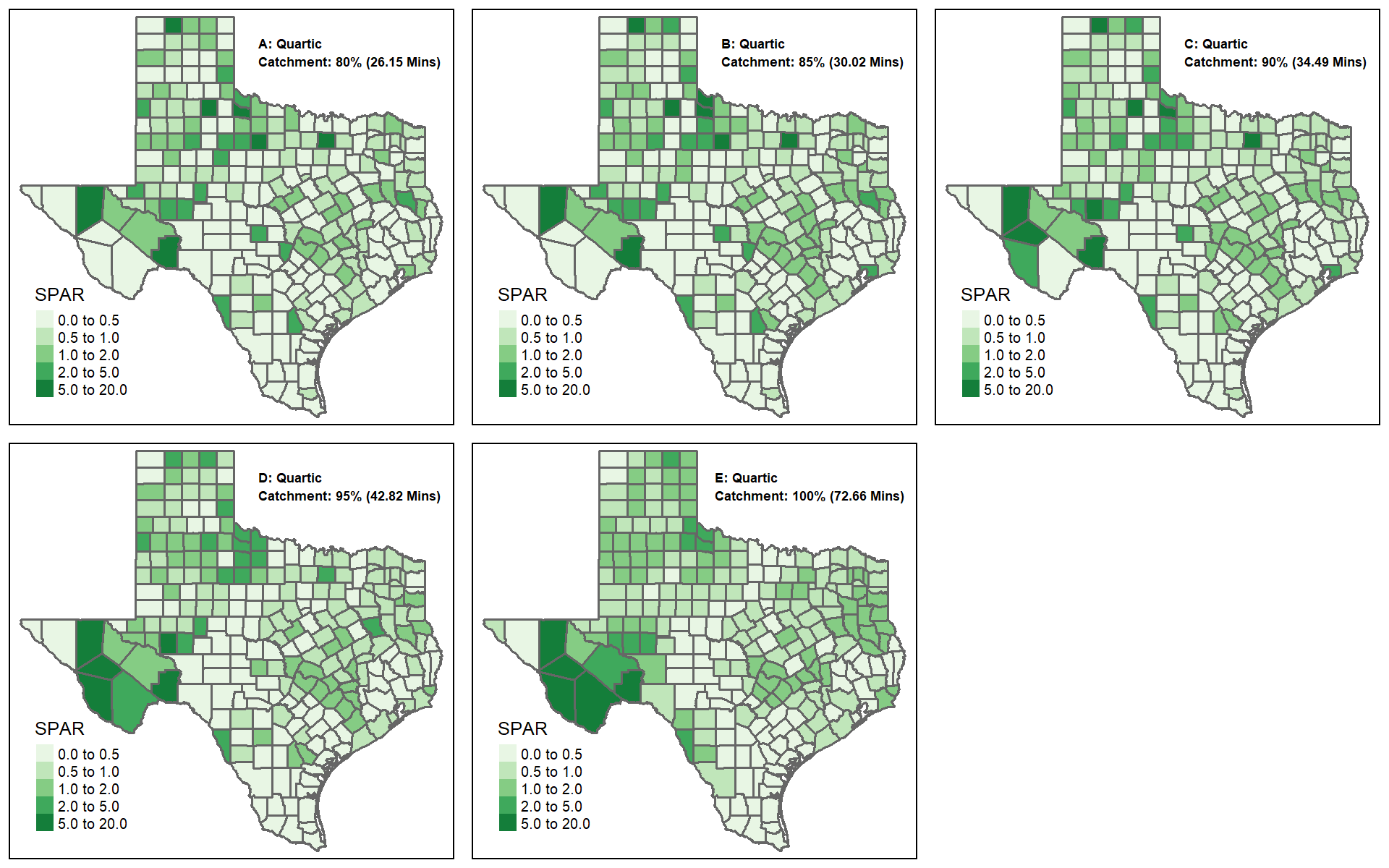


**Supplementary Figure 6**. County-level spatial accessibility ratio (SPAR) values for individual clinics using the Quartic kernel density distance decay function across 5 catchment areas: 80% population access (A), 85% access (B), 90% access (C), 95% access (D), and 100% access (E).


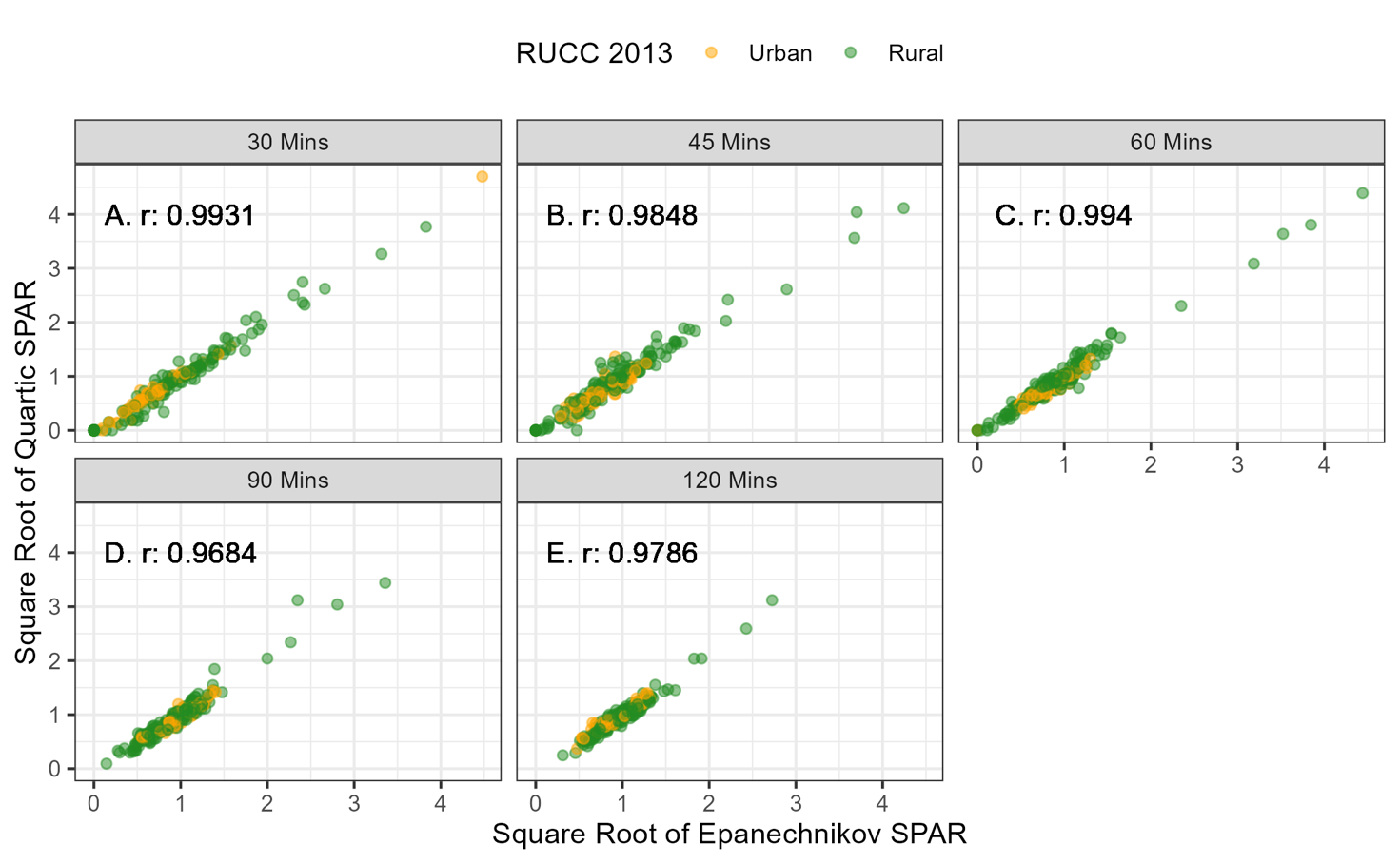


**Supplementary Figure 7**. Pearson correlation coefficients between the square root of Epanechnikov SPAR and Quartic SPAR for five catchments: 30 minutes (A), 45 minutes (B), 60 minutes (C), 90 minutes (D), and 120 minutes (E).


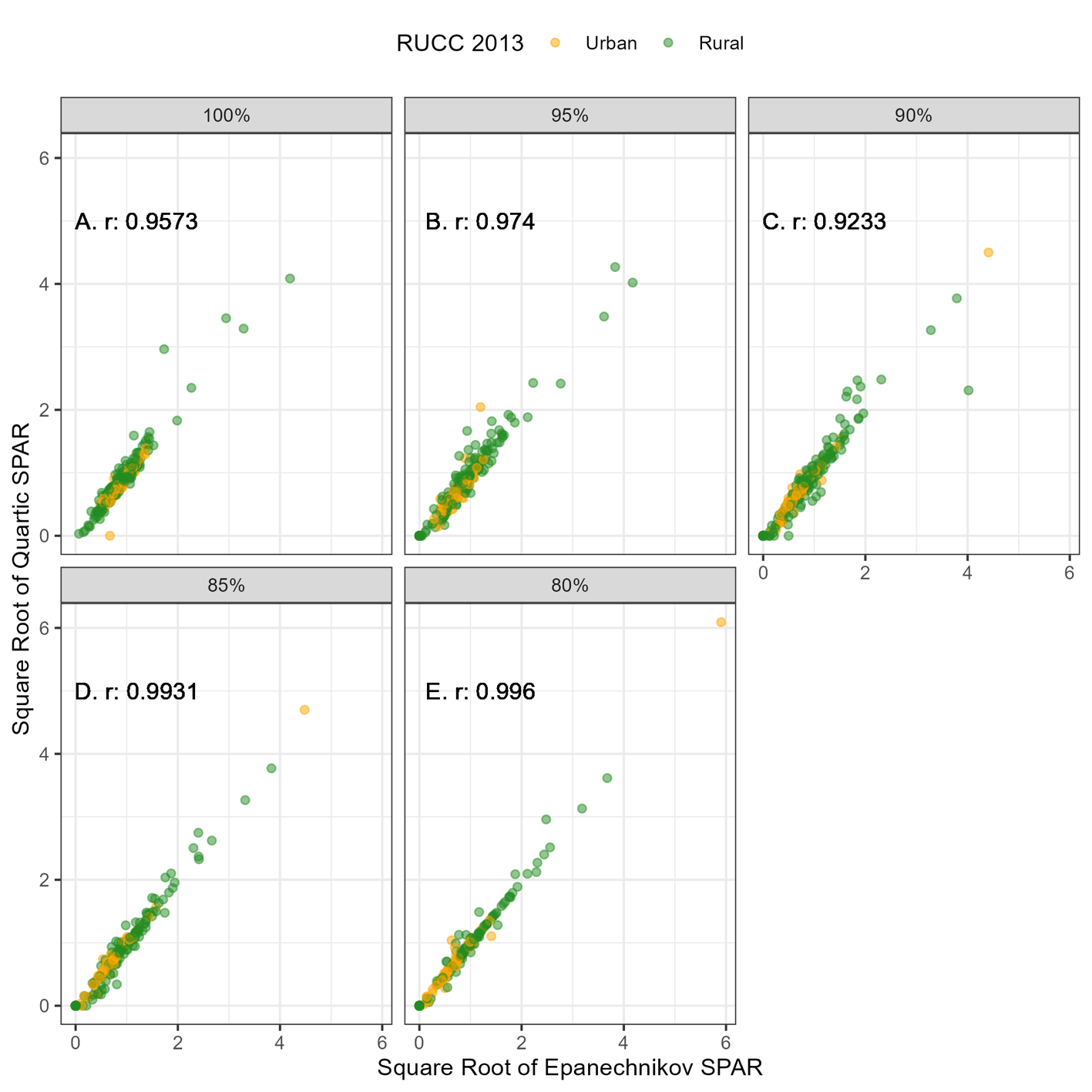


**Supplementary Figure 8**. Pearson correlation coefficient values between the square root of the Epanechnikov SPAR and Quartic SPAR for five catchments: 100% access (A), 95% access (B), 90% access (C), 85% access (D), and 80% access (E).

**Supplementary Table 1**. Pearson Correlation coefficients between SPAR values with the Epanechnikov decay function (E30-E120) and quartic decay function (Q30-Q120). Correlations were calculated across the 5 different time-based catchments: 30, 45, 60, 90, and 120 minutes.

|  | **Epanechnikov** | | | | | **Quartic** | | | | |
| --- | --- | --- | --- | --- | --- | --- | --- | --- | --- | --- |
|  | **E30** | **E45** | **E60** | **E90** | **E120** | **Q30** | **Q45** | **Q60** | **Q90** | **Q120** |
| **E30** | **1.000** |  |  |  |  |  |  |  |  |  |
| **E45** | 0.677*** | **1.000** |  |  |  |  |  |  |  |  |
| **E60** | 0.636*** | 0.975*** | **1.000** |  |  |  |  |  |  |  |
| **E90** | 0.190** | 0.662*** | 0.690*** | **1.000** |  |  |  |  |  |  |
| **E120** | 0.137* | 0.579*** | 0.592*** | 0.926*** | **1.000** |  |  |  |  |  |
| **Q30** | 0.990*** | 0.655*** | 0.611*** | 0.181** | 0.129* | **1.000** |  |  |  |  |
| **Q45** | 0.691*** | 0.985*** | 0.939*** | 0.634*** | 0.565*** | 0.675*** | **1.000** |  |  |  |
| **Q60** | 0.665*** | 0.991*** | 0.994*** | 0.679*** | 0.587*** | 0.641*** | 0.966*** | **1.000** |  |  |
| **Q90** | 0.321*** | 0.771*** | 0.798*** | 0.968*** | 0.847*** | 0.308*** | 0.741*** | 0.789*** | **1.000** |  |
| **Q120** | 0.136* | 0.599*** | 0.621*** | 0.961*** | 0.978*** | 0.128* | 0.576*** | 0.612*** | 0.886*** | **1.000** |

^1^ * p < 0.05, ** p < 0.01, *** p < 0.001
