# Supplementary material for: A comparison of floating catchment area parameters with applications to a dataset of clinics enrolled in a statewide child and adolescent psychiatric consultation program
Source: Front Public Health. 2025 Feb 20;13:1498819. doi: 10.3389/fpubh.2025.1498819 (PMC11882419; doi:10.3389/fpubh.2025.1498819)
Supplement: Supplementary file 2 [file Data_Sheet_1.pdf]

Code Template

Supplemental Material: A Comparison of Floating Catchment Area Parameters with Applications to a Dataset of Clinics Enrolled in a Statewide Child and Adolescent Psychiatric Consultation Program

Jocelyn Hunyadi, MPH   Lara S. Savas, PhD   Kehe Zhang, MS
Jeanette E. Deason, MPH   Ryan Ramphul, PhD   Melissa F. Peskin, PhD
Erica L. Frost, MPH   Cici Bauer, PhD, MS

2024-12-13

Contents

Data & Initial Processing **1**

Data Sources 1

Data Processing 2

OD Matrices **5**

Kernel-Density 2SFCA **5**

Determine Catchments 6

Distance Decay 6

Kernel Density 2SFCA 7

Data & Initial Processing

Data Sources

The following data sources used in this work are publicly available through download or through API queries:

1. **Census** - *Population & Geospatial Boundaries*. Query the Census API for geospatial boundaries for Texas at the county-level and acquire population data estimates from the *American Community Survey*, 2017-2021.
2. **Census** - *Population-Weighted County-Level Centroids*. Obtained for the 2020 Census and acquired at: https://www.census.gov/geographies/reference-files/time-series/geo/centers-population.html.
3. **CDC** - *Social Vulnerability Index* (SVI). Estimates from 2020 at the county-level. Data is publicly available at: https://www.atsdr.cdc.gov/placeandhealth/svi/data_documentation_download.html
4. **USDA** - *RUCC 2013 Rural Urban Codes.* Classified counties as *metro* (levels 1-3) or *nonmetro* (levels 4-9), which can be used to classify counties as *urban* or *rural*, respectively. The 2013 codes can be found at https://www.ers.usda.gov/data-products/rural-urban-continuum-codes.aspx

Zgodic et al. (2023) used small-area estimation to generate county-level estimates for ADHD prevalence among 5-17 year old children and adolescents at the regional, state, and county level in the United States. This publication provides the data at the county level in its supplementary material, providing a more fine-grain look at ADHD compared to utilizing the national-level or state-level estimates.

We used the Texas Child Psychiatry Access network (CPAN) data from the Texas Child Mental Health Care Consortium (TCMHCC). This dataset includes currently enrolled and participating clinics, including their geographical coordinates (longitude, latitude). *This dataset is NOT publicly available.*

Data Processing

Processing steps for the incorporated data are outlined below.

Query and Prepare Census Data

To query the Census API directly, a Census API key is required.

```
#### SET CENSUS API KEY ####
census_api_key("INSERT KEY HERE", install = T)
readRenviron("~/.Renviron")
  ## NOTE: Once key has been registered to the R environment,
  ## running this chunk may not be acquired
```

```
#### TEXAS - Counties ####
texas_counties <- get_acs(geography = "county", year = 2021, state = "TX",
                          variables = c("S0101_C01_001E", "S0101_C01_002E",
                                        "S0101_C01_003E", "S0101_C01_004E",
                                        "S0101_C01_005E", "S0101_C01_020E",
                                        "S0101_C01_021E", "S0101_C01_022E"),
                          geometry = T) %>%
  st_transform("+proj=longlat +datum=WGS84") %>%
  spread(key = variable, value = estimate) %>% dplyr::select(-moe) %>%
  rename(Total_pop = S0101_C01_001, Under5 = S0101_C01_002, Age5_9 = S0101_C01_003,
         Age10_14 = S0101_C01_004, Age15_19 = S0101_C01_005, Age5_14 = S0101_C01_020,
         Age15_17 = S0101_C01_021, Under18 = S0101_C01_022) %>%
  group_by(GEOID) %>%
  ## Fill in NA values and retain one row per County ##
  tidyr::fill(Total_pop, .direction = "updown") %>%
  tidyr::fill(Under5, .direction = "updown") %>%
  tidyr::fill(Age5_9, .direction = "updown") %>%
  tidyr::fill(Age10_14, .direction = "updown") %>%
  tidyr::fill(Age15_19, .direction = "updown") %>%
  tidyr::fill(Age5_14, .direction = "updown") %>%
  tidyr::fill(Age15_17, .direction = "updown") %>%
  tidyr::fill(Under18, .direction = "updown") %>%
  ungroup() %>%
  distinct(GEOID, .keep_all = T) %>%
  mutate(County = str_remove(NAME, " County, Texas"))
```

```r
## Save data to an appropriate location for future use
```

Additional Data Preparation

```r
#### LOAD AND CLEAN DATA ####
  ## Ensure Data is stored within an appropriate folder ##
  ## NOTE: Data must be initially loaded prior to running the following steps.

#### CPAN CLINIC DATA ####
clinic_dat <- clinic_dat %>% mutate(fips = as.character(fips)) %>%
  ## Get County Name of Clinic Location ##
  left_join(texas_counties[,c("GEOID", "County")], by = c("fips" = "GEOID")) %>%
  dplyr::select(-geometry) %>%
  dplyr::select(clinic_id, fips, County, clinic_name, latitude, longitude)


#### SVI DATA - 2020 ####
SVI.2020 <- SVI.2020 %>%
  rename(SVI1 = RPL_THEME1, SVI2 = RPL_THEME2, SVI3 = RPL_THEME3,
         SVI4 = RPL_THEME4, SVI = RPL_THEMES) %>%
  dplyr::select(STCNTY, COUNTY, FIPS, AREA_SQMI, contains("SVI"))


#### RURAL/URBAN CLASSIFICATION - RUCC 2013 ####
RUCC.2013 <- RUCC.2013 %>% filter(State == "TX") %>%
  mutate(RUCC_Urban_2013 = ifelse(RUCC_2013 %in% c(1,2,3), 1, 0),
         RUCC_Urban_2013 = factor(RUCC_Urban_2013, levels = c(1, 0),
                                  labels = c("Urban", "Rural"))) %>%
  mutate(County = str_remove(County_Name, " County")) %>%
  dplyr::select(FIPS, County, RUCC_Urban_2013)


#### POPULATION-WEIGHTED CENTROIDS 2020 ####
Pop.centroid <- Pop.centroid %>%
  mutate(COUNTYFP = as.character(COUNTYFP), STATEFP = as.character(STATEFP)) %>%
  mutate(FPS = ifelse(str_length(COUNTYFP) == 1, paste0(STATEFP, "00", COUNTYFP),
                      ifelse(str_length(COUNTYFP) == 2,
                             paste0(STATEFP, "0", COUNTYFP),
                             paste0(STATEFP, COUNTYFP)))) %>%
  dplyr::select(FPS, COUNAME, LATITUDE, LONGITUDE) %>%
  dplyr::rename(County = COUNAME, Latitude_PW = LATITUDE,
                Longitude_PW = LONGITUDE)


#### MERGE ####
  ## Working County-Level Dataframe ##
County.Merge <- texas_counties %>%
  mutate(Age5_17 = Age5_14 + Age15_17) %>%
  dplyr::select(GEOID, County, Total_pop, Under18, Age5_17, geometry) %>%
```

```
  left_join(SVI.2020, by = c("County" = "COUNTY")) %>%
  dplyr::select(-STCNTY, -FIPS) %>%
  left_join(RUCC.2013, by = "County") %>% dplyr::select(-FIPS) %>%
  left_join(Pop.centroid, by = c("County")) %>% dplyr::select(-FPS)

## Save data as appropriate.
```

County-Level ADHD

```
#### ZGODIC ET AL 2023 ADHD PREVALENCE ####
  ## Ensure data is loaded previously.
  ## Filter for Texas Only ##
ADHD.df <- rADHD.df %>%
  filter(State == "TX") %>%
  ## Separate Estimate and CI Bounds ##
  mutate(ADHD_Est = str_remove(`ADHD Prevalence Estimate`,
                               "\\([0-9]+[:punct:]?[0-9]*-[0-9]+[:punct:]?[0-9]*\\)"),
         ADHD_L95CI = str_remove(str_remove(str_extract(`ADHD Prevalence Estimate`,
                                                         "\\([0-9]+[:punct:]?[0-9]*-"),
                                             "\\("), "-"),
         ADHD_U95CI = str_remove(str_remove(str_extract(`ADHD Prevalence Estimate`,
                                                         "-[0-9]+[:punct:]?[0-9]*\\)"),
                                             "\\)"), "-")) %>%
  rename(ADHD_Est_CI = `ADHD Prevalence Estimate`) %>%
  mutate(ADHD_Est = as.numeric(ADHD_Est),
         ADHD_L95CI = as.numeric(ADHD_L95CI),
         ADHD_U95CI = as.numeric(ADHD_U95CI))

  ## Check DeWitt County ##
ADHD.df$County[which(ADHD.df$County == "De Witt")] <- "DeWitt"


#### MERGE TO COUNTY DATA ####
ADHD.df2 <- ADHD.df %>% dplyr::select(County, contains("ADHD"))
County.Merge <- County.Merge %>% left_join(ADHD.df2, by = "County")
```

OD Matrices

Prior to performing 2SFCA, we first constructed the **Origin-Destination** (OD) matrix, which is designed to quantify the travel time from a given demand (origin) point i (e.g., population-weighted county centroid) to a supply (destination) point j (e.g., CPAN clinic). Each cell within the OD matrix represents a unique origin-destination pair. The resulting OD matrix is of J by I dimensions, where J is the number of destinations points and I is the number of origin points.

Travel duration between each unique pair was completed using ArcGIS Pro (v. 3.1.1) with the StreetMap Premium extension. In ArcGIS, *driving time* was specified as the mode with no cutoff so that all driving times in the matrix were returned.

Kernel-Density 2SFCA

```
#### LOAD PREVIOUSLY PROCESSED FILES ####
  ## County Data with SVI ##
County.df <- County.merge %>%
  ## Create population of youth (5-17) diagnosed with ADHD
  mutate(ADHD_Demand = trunc(Age5_17*(ADHD_Est/100)))

  ## CPAN Clinic Location ##
clinic_dat <- clinic_dat %>% filter(!is.na(County))
clinic.sf <- clinic_dat %>% st_as_sf(coords = c("longitude", "latitude"),
                                     crs = "+proj=longlat +datum=WGS84")

  ## US City Coordinates ##
  ## From the maps package ##
us.df <- maps::us.cities %>% filter(country.etc == "TX") %>%
  arrange(desc(pop)) %>% dplyr::select(-country.etc, -capital) %>%
  rename(City = name) %>%
  mutate(City = str_remove(City, " TX$"))

  ## Select Top 10 Cities ##
us.10 <- us.df %>% slice(1:6)

  ## Convert to ShapeFile ##
us.sf <- us.10 %>% st_as_sf(coords = c("long", "lat"),
                            crs = "+proj=longlat +datum=WGS84")


#### DISTANCE MATRICES ####
  ## Complete Distance Matrices were generated in ArcGIS ##
OD_Complete <- OD_Complete %>%
  separate(CountyToCPAN, sep = " - ", into = c("County", "Clinic"))
```

Determine Catchments

One approach to defining the maximum catchment area is to simply specify times: (1) 30 minutes, (2) 45 minutes, (3) 60 minutes, (4) 90 minutes, and (5) 120 minutes.

An alternative approach is to define the catchment area based on the population that has access to *at least CPAN clinic*. We selected the following percentages: 100%, 95%, 90%, 85%, and 80%.

```
#### SET UP ####
catch100 <- c()
catch95 <- c()
catch90 <- c()
catch85 <- c()
catch80 <- c()

#### NEAREST ####
  ## Nearest CPAN clinic for each County ##
  ## Based on population-weighted centroid ##
OD_Nearest <- OD_Complete %>%
  arrange(County, Total_TravelTime) %>%
  group_by(County) %>%
  slice(1)

#### Times Associated with Percent Thresholds ####
Complete100 <- max(OD_Nearest$Total_TravelTime) + 1 ## 71.66 minutes
Quant_Comp <- quantile(OD_Nearest$Total_TravelTime,
                       probs = c(0.8, 0.85, 0.9, 0.95))
Complete95 <- Quant_Comp[[4]] + 0.5
Complete90 <- Quant_Comp[[3]] + 0.5
Complete85 <- Quant_Comp[[2]] + 0.5
Complete80 <- Quant_Comp[[1]] + 0.5
```

Distance Decay

```
#### EPANECHNIKOV KERNEL ####
Kweight <- function(Duration, max) {
  d_h <- (Duration/max)^2
  weight <- 0.75*(1-d_h)
  weight <- round(weight, 2)
  return(weight)
}

#### QUARTIC KERNEL ####
Qweight <- function(Duration, max) {
  init <- 15

  ## GENERATE WEIGHT ##
  if(Duration < init) {
    weight <- 1
```

```
  } else if (Duration >= init & Duration <= max) {
    weight <- (15/16)*(1-(Duration/max)^2)^2
    weight <- round(weight, 2)
  } else {
    weight <- 0
  }

  ## RETURN ##
  return(weight)
}
```

Kernel Density 2SFCA

For the application of the *Quartic kernel*, the initial distance threshold is defined as **15 minutes**.

Weight Matrix

```
#### Apply the distance decay functions to each cell of the OD matrix ####

#### INITIALIZE ####
  ## Vector of Catchment ##
Comp_catch <- c(30, 45, 60, 90, 120, Complete100, Complete95, Complete90,
                Complete85, Complete80)

  ## Initialize ##
EP.Wt.Comp <- list()
QU.Wt.Comp <- list()


#### (1) EPANECHNIKOV LOOP ####
for(i in 1:length(Comp_catch)) {
  ## Apply the Epanechnikov Kernel ##
  EP.Wt.Comp[[i]] <- OD_Complete %>% dplyr::select(Clinic, County, Total_TravelTime) %>%
    spread(key = County, value = Total_TravelTime) %>%
    mutate(across(Anderson:Zavala, Kweight, max = Comp_catch[i])) %>%
    mutate(across(Anderson:Zavala, ~ifelse(.x < 0, 0, .x)))

}

#### (2) QUARTIC LOOP ####
for(j in 1:length(Comp_catch)) {
  ## Apply the Quartic Kernel ##
  hold <- OD_Complete %>% dplyr::select(Clinic, County, Total_TravelTime) %>%
    spread(key = County, value = Total_TravelTime)

  for(l in 2:ncol(hold)) {
    ## APPLY ##
    for(m in 1:nrow(hold)) {
      hold[m,l] <- Qweight(Duration = hold[m,l], max = Comp_catch[j])
```

```
    }
  }

  QU.Wt.Comp[[j]] <- hold
}


#### MAKE MATRIX ####
EP.Wt.Comp.m <- data.matrix(EP.Wt.Comp, rownames.force = NA)
QU.Wt.Comp.m <- data.matrix(QU.Wt.Comp, rownames.force = NA)
```

Demand Vector

NOTE: Ensure that the main dataframe (*County.merge*) is arranged **alphabetically** to ensure that the demand vector matches the weight matrices for proper matrix multiplication.

```
#### DEMAND VECTOR ####
texas_demand <- County.merge %>% as.data.frame() %>%
  dplyr::select(County, ADHD_Demand) %>%
  arrange(County)

#### TRANSPOSE ####
pop_vector <- t(as.data.frame(as.numeric(texas_demand$ADHD_Demand)))
colnames(pop_vector) <- texas_demand$County
rownames(pop_vector) <- "Population"
```

Supply Vector

```
#### Multiply the OD matrix by the demand of each county ####

#### INITIALIZE ####
EP.Comp.Prov <- list()
QU.Comp.Prov <- list()


#### CREATE PROVIDER VECTORS ####
for(i in 1:length(EP.Wt.Comp.m)) {
  EP.Comp.Prov[[i]] <- pop_vector %*% t(EP.Wt.Comp.m[[i]][,-c(1)])
}

for(k in 1:length(QU.Wt.Comp.m)) {
  QU.Comp.Prov[[k]] <- pop_vector %*% t(QU.Wt.Comp.m[[k]][,-c(1)])
}
```

Supply-to-Demand Ratio

```
#### CREATE SUPPLY FOR PROVIDER ####
supply <- 1
  ## In the absence of clinic capacity data, consider only presence of supply pt.


#### CREATE SUPPLY-DEMAND RATIO PER 100,000 RESIDENTS ####
for(i in 1:length(EP.Comp.Prov)) {
  ## For all 10 in List ##
  cur <- EP.Comp.Prov[[i]]
  cur2 <- QU.Comp.Prov[[i]]

  ## LOOP THROUGH DF ##
  for(k in 1:ncol(cur)) {
    if(cur[1,k] != 0) {
      cur[1,k] <- ((100000 * supply) / cur[1,k])
    }

    if(cur2[1,k] != 0) {
      cur2[1,k] <- ((100000 * supply) / cur2[1,k])
    }
  }

  EP.Comp.Prov[[i]] <- cur
  QU.Comp.Prov[[i]] <- cur2

}
```

Spatial Accessibility Index (SPAI) and Spatial Accessibility Ratio (SPAR)

```
#### Apply the Distance Decay Function to the Second Step ####

#### INITIALIZE ####
EP.Comp.SPAI <- list()
QU.Comp.SPAI <- list()

for(i in 1:length(EP.Comp.Prov)) {
  #### APPLY DISTANCE DECAY IN STEP 2 ####
  hold <- EP.Comp.Prov[[i]] %*% as.matrix(EP.Wt.Comp.m[[i]][,-c(1)])
  hold2 <- QU.Comp.Prov[[i]] %*% as.matrix(QU.Wt.Comp.m[[i]][,-c(1)])

  #### FORMAT SPAI VALUES & CALCULATE SPAR ####
  hold <- as.data.frame(t(hold)) %>%
    rename(SPAI = Population) %>% rownames_to_column(var = "County") %>%
    mutate(SPAR = SPAI/mean(SPAI)) %>% dplyr::select(County, SPAI, SPAR) %>%
    mutate(Catchment = Comp_catch[[i]],
           Dist_Decay = "Epanechnikov")
  hold2 <- as.data.frame(t(hold2)) %>%
```

```
    rename(SPAI = Population) %>% rownames_to_column(var = "County") %>%
    mutate(SPAR = SPAI/mean(SPAI)) %>% dplyr::select(County, SPAI, SPAR) %>%
    mutate(Catchment = Comp_catch[[i]],
           Dist_Decay = "Quartic")

  #### PUT IN LIST ####
  EP.Comp.SPAI[[i]] <- hold
  QU.Comp.SPAI[[i]] <- hold2

}
```

```
#### MERGE WITH COUNTY ####
EP.Comp.SPAR <- list()
QU.Comp.SPAR <- list()

merger.df <- County.merge %>% mutate(County = as.character(County))

for(i in 1:length(EP.Comp.SPAI)) {
  EP.Comp.SPAR[[i]] <- merger.df %>% left_join(EP.Comp.SPAI[[i]], by = "County")
  QU.Comp.SPAR[[i]] <- merger.df %>% left_join(QU.Comp.SPAI[[i]], by = "County")
}
```

Compile Final Output

```
#### Time Thresholds ####
Comp.SPAR1 <- rbind(EP.Comp.SPAR[[1]], EP.Comp.SPAR[[2]], EP.Comp.SPAR[[3]],
                    EP.Comp.SPAR[[4]], EP.Comp.SPAR[[5]], QU.Comp.SPAR[[1]],
                    QU.Comp.SPAR[[2]], QU.Comp.SPAR[[3]],
                    QU.Comp.SPAR[[4]], QU.Comp.SPAR[[5]]) %>%
  mutate(Catchment = case_when(
    Catchment == 30 ~ "30 Mins",
    Catchment == 45 ~ "45 Mins",
    Catchment == 60 ~ "60 Mins",
    Catchment == 90 ~ "90 Mins",
    Catchment == 120 ~ "120 Mins",
    TRUE ~ NA
  )) %>%
  mutate(Catchment = factor(Catchment, levels = c("30 Mins", "45 Mins", "60 Mins",
                                                  "90 Mins", "120 Mins")))


#### Percent Access Thresholds ####
Comp.SPAR2 <- rbind(EP.Comp.SPAR[[6]], EP.Comp.SPAR[[7]], EP.Comp.SPAR[[8]],
                    EP.Comp.SPAR[[9]], EP.Comp.SPAR[[10]], QU.Comp.SPAR[[6]],
                    QU.Comp.SPAR[[7]], QU.Comp.SPAR[[8]], QU.Comp.SPAR[[9]],
                    QU.Comp.SPAR[[10]]) %>%
  mutate(Catchemnt = case_when(
    Catchment > 70 ~ "100%",
    Catchment < 70 & Catchment > 40 ~ "95%",
```

```
    Catchment < 40 & Catchment > 32 ~ "90%",
    Catchment < 32 & Catchment > 28 ~ "85%",
    Catchment < 28 ~ "80%",
    TRUE ~ NA
  )) %>%
  mutate(Catchment = factor(Catchment, levels = c("100%", "95%", "90%",
                                                  "85%", "80%")))
```
